# Preliminary Efficacy of a Digital Intervention for Adolescent Depression: Randomized Controlled Trial

**DOI:** 10.2196/48467

**Published:** 2024-02-07

**Authors:** Emily Peake, Ian Miller, Jessica Flannery, Lang Chen, Jessica Lake, Aarthi Padmanabhan

**Affiliations:** 1 Limbix Health Inc San Francisco, CA United States; 2 Big Health Inc San Francisco, CA United States; 3 Digital Medicine Society Boston, MA United States; 4 Akili Boston, MA United States; 5 Neuroscience Unit Santa Clara University Santa Clara, CA United States

**Keywords:** adolescent, depression, randomized controlled trial, mobile phone, digital therapeutics, mobile app, cognitive behavioral therapy, behavioral activation, mobile health

## Abstract

**Background:**

Adolescent depression is a significant public health concern; however, access to effective mental health care is limited. Digital therapeutics (DTx) can improve access to evidence-based interventions; however, their efficacy in adolescents is sparsely documented.

**Objective:**

This study aims to examine the efficacy of a mobile app DTx versus an active control as an adjunct treatment for adolescent depression symptoms.

**Methods:**

An internet-based open-label randomized control trial was conducted nationwide with a partial crossover design, and 168 adolescents aged 13 to 21 years with symptoms of depression were recruited between November 2020 and September 2021. Participants were randomized (1:1) to the cognitive behavioral therapy–based treatment app (Spark) or to a psychoeducational control app (control), which they would use for a duration of 5 weeks. The primary outcome was a between-group (Spark vs control) difference in the change in depression symptoms from baseline to postintervention, as measured by the Patient Health Questionnaire-8 (PHQ-8) using a linear mixed-effects analysis. The PHQ-8 ranges from 0 to 24, with scores of 5 to 9 indicating mild depression symptoms, scores of 10 to 14 indicating moderate symptoms, scores of 15 to 19 indicating moderately severe symptoms, and scores of 20 to 24 indicating severe symptoms. A minimal clinically important difference (5-point reduction between baseline and postintervention) in the Spark arm and group differences in remission and treatment response rates based on the PHQ-8 at postintervention were also investigated.

**Results:**

A total of 160 participants were randomized, 80 in the Spark arm (mean age 16.89, SD 2.5 y) and 80 in the control arm (mean age 16.79, SD 2.59 y). Data from 121 participants (Spark: n=63; control: n=58) with moderate to severe (PHQ-8≥10) symptoms at baseline were included in the primary analyses following a modified intention-to-treat principle. A linear mixed-effect analysis revealed a nonsignificant difference between the study arms in depression symptom change over the intervention period. The Spark arm met a minimal clinically important difference threshold (mean −5.08, 95% CI −6.72 to −3.42). The remission rate in the Spark arm was significantly higher than that in the control arm (11/63, 17% vs 2/58, 3%; *χ*^2^_1_=6.2; *P*=.01; false discovery rate–adjusted *P*=.03). The treatment response rates were not significantly different between the study arms (*P*=.07; false discovery rate–adjusted *P*=.16). Post hoc analyses including participants with mild to severe (PHQ-8 score ≥5) symptoms at baseline revealed promising evidence that Spark is effective in those with mild to severe symptoms.

**Conclusions:**

There is initial evidence that a self-guided, cognitive behavioral therapy–based DTx intervention may effectively treat mild to severe depression symptoms in adolescents. DTx may improve access to mental health care for adolescents or serve as an important adjunct to the standard of care.

**Trial Registration:**

ClinicalTrials.gov NCT04524598; https://clinicaltrials.gov/study/NCT04524598

## Introduction

### Background

Depression is a pervasive mental health disorder affecting approximately 4 million adolescents in the United States, and this number is expected to grow annually [1-4]. Depression can be lifelong, often emerging in adolescence and increasing the risk of poor physical health, social and relational problems, academic and employment difficulties, and reduced overall well-being [5-7]. Despite a growing need for effective treatment options, adolescents access [3,6] and adhere to [8,9] mental health treatment at alarmingly low rates owing to a number of factors. Stigma and a general difficulty in identifying symptoms of a mental illness inhibit adolescents’ willingness or awareness of seeking treatment [10,11]. When they do seek help, adolescents face months-long waitlists [12]. Other external forces drive the unmet clinical needs of adolescents experiencing depression, including costs [2,13], a widening divide in the accessibility of treatment [14,15], and an outstripped supply of mental health professionals [14,16]. Barriers in access to mental health treatment and depression incidence rates in adolescents have been exacerbated by the COVID-19 pandemic, necessitating action and new solutions [17-21].

Digital health programs, accessible through smartphones and computers, offer potential avenues for delivering treatment options for mental health conditions, such as depression [22-29]. However, it is essential to acknowledge their limitations, which include the absence of evidence-based protocols and clinical validation [24,25,27]. Digital therapeutics (DTx), which are evidence-based software programs designed to prevent, treat, or manage health conditions, serve to address these limitations [30]. Smartphone-based DTx are particularly well suited for adolescents given their technological literacy, ubiquitous cell phone use, and privacy preferences [22,29,31,32]. DTx are also easily scalable, can serve as adjunct to standard care, improve treatment fidelity [33-35], are cost-effective [36]. These technologies have been shown to be preliminarily effective in treating adolescents with a variety of mental health concerns including attention-deficit/hyperactivity disorder [37], sleep disorders, eating disorders, and anxiety [38]. Ultimately, DTx fill critical gaps in non-DTx digital treatments and can help patients receive effective, data-driven treatment with the added benefit of home access at any time [35,39,40].

Cognitive behavioral therapy (CBT) is a widely recognized and recommended treatment for depression and is considered the standard of care for adolescents [41-43]. Behavioral activation (BA), an important component of CBT [44-47], involves engaging in value-based behaviors that are rewarding or elicit a sense of mastery to help reduce depression symptoms using a combination of motivational strategies, reward seeking, and natural reinforcers, alongside reducing maladaptive and avoidant behaviors. BA has been shown to be an effective stand-alone treatment for adolescent depression [44-48]. Digital CBT applications have demonstrated promise in treating depression [23,26,49,50], and a BA-focused digital therapeutic may represent an effective and accessible treatment option for adolescents.

Limitations such as low user engagement and insufficient existing clinical validation (eg, randomized controlled trials [RCTs]) may limit the widespread adoption of DTx [24,25,27,49]. To our knowledge, there are no DTx for depression tailored specifically for adolescents, including BA-based DTx, and limited evidence exists regarding the efficacy of a smartphone-based DTx for adolescent depression [51-53].

### Objectives

This study evaluates the efficacy of Spark*,* a 5-week self-guided mobile app intervention based on validated BA treatment protocols for adolescents with depression [44,45]. Adolescents aged 13 to 21 years were recruited nationwide into an internet-based RCT comparing Spark with an active control. Our main goal was to assess the preliminary efficacy of Spark in treating moderate to severe depression symptoms as an adjunct to usual care by measuring group differences in (1) changes in depression symptoms over time, including whether each group had a MCID in depression symptoms; (2) the proportion of participants who achieved remission; and (3) the proportion of participants who demonstrated treatment response. As this RCT was conducted between November 2020 and September 2021, another goal was to provide mental health resources to adolescents experiencing symptoms of depression during the COVID-19 pandemic.

## Methods

### Study Design

This open-label, partial crossover RCT compared Spark with an app with psychoeducational content (control app) in adolescents aged 13 to 21 years with self-identified depression symptoms. The study was conducted in 2 phases: an evaluation of feasibility (phase 1) and a preliminary evaluation of efficacy (phase 2). The results from phase 1 (Spark version 2.0) have been reported elsewhere [54]. Here, we focus on the results from phase 2, which combined data collected using Spark version 2.1 and version 2.2 (refer to the Spark section for more information on the versions). Updates to the Spark app to version 2.2 were deployed approximately halfway during the study; version 2.1 was used between September 2020 and March 2021, and version 2.2 was used between June 2021 and September 2021. The study design, randomization, control app, and outcome measures were the same across both versions. There was an a priori plan to combine the data from the 2 Spark versions for analysis.

### Ethical Considerations

The study was approved by the Institutional Review Board of the Western Copernicus Group (20201686) under a nonsignificant risk investigational device exemption and overseen by an independent data and safety monitoring board. The trial was also registered on ClinicalTrials.gov (NCT04524598).

### Participants

Participants were recruited nationwide via internet-based advertising and word of mouth. The following inclusion criteria were applied in phase 2: (1) aged 13 to 21 years, (2) self-reported symptoms of depression, (3) residing in the United States for the duration of the 5-week study, (4) under the care of a US-based primary care or licensed mental health care provider and willing and able to provide the name and contact information of the provider during consent appointment, (5) English fluency and literacy of adolescents and consenting legal guardians if aged <18 years, (6) access to a smartphone (iPhone 5s or later or running Android 4.4 KitKat or later) and regular internet access, (7) willingness to provide informed e-consent or assent and have legal guardian willing to provide informed e-consent if aged >18 years, and (8) stable for at least 2 months on any treatment (including medication or psychotherapy) for a mental health disorder.

Participants were excluded if they self-reported any of the following: (1) self-reported lifetime suicide attempt or active self-harm or active suicidal ideation with intent; (2) diagnosed by a clinician with bipolar disorder, substance use disorder, or any psychotic disorder including schizophrenia; (3) incapable of understanding or completing study procedures and digital intervention as determined by the participant, patient or legal guardian, health care provider, or clinical research team, and (4) previously participated in the user testing or clinical testing of the Spark app.

The legal guardians provided consent for participants aged <18 years. Adolescent participants provided consent unless they were under 18. If they were under 18, their legal guardians provided consent and they provided assent.

### Procedure

During a virtual consent and enrollment session over video conferencing, participants and their legal guardians (if aged <18 y) were provided with study details, provided electronic informed consent or assent, were assessed for eligibility, and completed web-based baseline questionnaires. Participants downloaded an app on their smartphone and were provided with a safety plan template with instructions [55] to complete it on their own as a personal resource. Participants were randomized 1:1 to the Spark or control arm using a block randomization approach ranging in multiples of 2 from 6 to 12 [56].

All participants had access to their assigned app for the 5-week intervention period. During the intervention period, participants completed an in-app Patient Health Questionnaire-8 (PHQ-8) [57], a self-report questionnaire to assess symptoms of depression, and an internally developed symptom check questionnaire weekly, which they could complete anytime over a 7-day period. Legal guardians completed a weekly symptom check about their child on the web. Following the 5-week intervention period, app access was restricted, and participants and legal guardians completed web-based postintervention questionnaires. Participants randomized to the control arm were also offered access to the Spark app after the 5-week intervention period and completed postintervention questionnaires on the web again following this second intervention period (partial crossover data not reported here). Baseline and postintervention measures assessed participant characteristics, concurrent treatments, depression and anxiety symptoms, resilience, app feedback, and impacts of COVID-19 (refer to Table 1 for schedule and full descriptions) [57-63].

**Table 1:** Schedule of assessments and descriptions.

Assessment name	Baseline	Week 1	Week 2	Week 3	Week 4	Week 5 postintervention	Description
Baseline questionnaire^a^^*,*^^b^	✓						An internally developed self-report and legal guardian–report questionnaire to collect gender, age, ethnicity, race, education and parent or legal guardian education, medication use, previous and current treatment for mental health disorders, depression diagnosis status, other mental or neurological disorder diagnosis status, and positive and negative impacts of COVID-19.
Brief Resilience Scale^a^ [58]	✓						A standardized 6-item self-report measure for assessing the ability to recover from stress. Each item is rated on a frequency scale from 1 (strongly disagree) to 5 (strongly agree).
Patient Health Questionnaire-8^a^ [57]	✓	✓	✓	✓	✓	✓	A standardized 8-item self-report measure of depression symptom severity over the past 2 weeks. Each item is rated on a frequency scale from 0 (never) to 3 (nearly every day).
Weekly symptom check^a^^*,*^^b^	✓	✓	✓	✓	✓	✓	An internally developed self-report and legal guardian–report questionnaire to collect information on any negative symptoms or side effects experienced during the intervention period, the severity of each event, and its relatedness to the mobile app.
Generalized Anxiety Disorder-7^a^ [59]	✓					✓	A standardized 7-item self-report measure of anxiety symptom severity over the past 2 weeks. Each item is rated on a frequency scale from 0 (never) to 3 (nearly every day).
PROMIS^c^ Pediatric Global 7+2^a^ [60]	✓					✓	A standardized 7+2–item self-report measure that produces a unidimensional measure of global health perception or well-being and separate measures of fatigue and pain. Each item is rated on a frequency scale from 1 to 5.
PROMIS Pediatric Global 7+2 Parent Proxy^b^ [60]	✓					✓	A standardized 7+2–item legal guardian–report measure that produces a unidimensional measure of global health perception or well-being and separate measures of fatigue and pain. Each item is rated on a frequency scale from 1 to 5.
Mood and Feelings Questionnaire Parent Form^b^ [61]	✓					✓	A standardized 13-item legal guardian–report measure to assess how the participant has been feeling or acting over the past 2 weeks. Each item is rated on a frequency scale from 0 (not true) to 2 (true).
System usability scale^a^ [62]						✓	A standardized 10-item self-report measure on the usability of systems such as computer programs or mobile apps, including the perceived ease of use, complexity, and consistency of systems. Each item is rated on a frequency scale of 1 (strongly disagree) to 5 (strongly agree).
User engagement scale-short form^a^ [63]						✓	A standardized 12-item self-report measure on 4 specific engagement factors, including focused attention, perceived usability, esthetic appeal, and reward. Each item is rated on a frequency scale of 1 (strongly disagree) to 5 (strongly agree).
Postintervention questionnaire^a^						✓	An internally developed self-report and legal guardian–report questionnaire to collect information regarding any change in concurrent treatment for depression during the study, mood or depression symptom improvement and enjoyment associated with the app, general feedback about the app or research participation, and positive and negative impacts of COVID-19 since baseline.

^a^Completed by adolescent.

^b^Completed by caregiver.

^c^PROMIS: Patient-Reported Outcomes Measurement Information System.

### Interventions

Both the Spark and control programs were divided into 5 modules recommended to be completed at a pace of 1 module per week over the 5-week intervention period, but the participants were able to progress at their own pace. Content for a given week was not expected to take >60 minutes to complete. All participants were prompted to complete a weekly PHQ-8 and a clinical concerns questionnaire on the mobile app. Automated app notification reminders to complete these questionnaires were sent. If users had not opened the app in 3 days, an automated app notification encouraging participants to use the app was sent. Automated reminder notifications were sent 7 days before the end of the intervention period to remind participants that the intervention period would be ending in 7 days. Crisis resources were available for participants to access anytime in each app.

### Spark (Versions 2.1 and 2.2)

The Spark app is based on CBT, which implements BA [44,45]. A character called “Limbot” is used as a therapeutic guide to encourage users to complete activities and model examples of activities for users. In the app, participants read text, answered questions, inputted text, and completed interactive activities. Participants were encouraged to schedule activities to be completed outside of the app and reflect on the impact on their mood. Tasks in the mobile app progress in a linear fashion, that is, each task must be completed to progress to the next task.

Version 2.1 of Spark included a 5-level program focused on providing psychoeducational content and delivering the BA model of depression by teaching 2 core skills (mood activity logging and activity scheduling). Version 2.2 was also divided into 5 levels and expanded on the content of version 2.1, adding problem-solving, mindfulness, and relapse prevention content. Version 2.2 also included a reward system and animations for completing certain activities, some design changes to the user interface, and an increased number of in-app notifications. The 5 levels of Spark version 2.1 were as follows:

- Level 1 (Start Your Journey): Program introduction and learning about the BA model of depression
- Level 2 (Making Choices): Mood tracking and up and down activities
- Level 3 (Solving Problems): Learn about activity scheduling and complete 3 activations,
- Level 4 (Staying Active): Complete 4 activations and
- Level 5 (Journey’s End): Complete 5 activations.

Version 2.2 expanded on version 2.1 with the addition of new features, UI elements, and content. The 5 levels of Spark version 2.2 were as follows:

- Level 1: Onboarding and Introduction to BA (no differences)
- Level 2: Mood tracking (no differences)
- Level 3: Mindfulness and Activity Scheduling (addition of 2 psychoeducational tasks teaching and reinforcing mindfulness skills)
- Level 4: Problem-Solving and Activity Scheduling (addition of 2 psychoeducational tasks teaching and reinforcing problem-solving skills), and
- Level 5: Relapse Prevention and Activity Scheduling (addition of 6 psychoeducational tasks teaching relapse prevention skills).

### Psychoeducational Control

The control app contained 5 modules of age-appropriate psychoeducational content related to the neurobiology of depression and did not contain any active CBT or BA components. In this app, participants read the text on screen related to the brain and behavior, the adolescent brain, depression in the brain, neurobiological factors influencing depression, and personality. The same version of the control app was used for the entire study. The 5 levels of the control app included Lesson 1: Understanding Behavior, Lesson 2: Exploring the Brain, Lesson 3: Mastering Messengers, Lesson 4: Riding the Wave, and Lesson 5: People and Personality.

### Safety Monitoring

A rigorous safety monitoring and classification procedure was followed. Potential safety-related events (ie, clinical concerns), which included worsening or persistently high depressive symptoms, any imminent risk events, suicidal ideation, hospitalizations, injury, illness, nonsuicidal self-injury, and direct or indirect indications of abuse, were identified in the following ways: (1) any concerning information provided to study staff during the onboarding session, in email, text, or phone correspondence during participation; (2) symptom deterioration based on weekly PHQ-8 scores was defined as (a) PHQ-8 score that was ≥15 and ≥5 points higher than the baseline score or (b) PHQ-8 scores ≥20 for 2 weeks in a row during the intervention, and (3) during the 5 week intervention period, all participants and their legal guardians (for those aged >18 y) were asked to complete a weekly internally developed symptom check questionnaire that asked them to report any negative symptoms or side effects that they experienced over the past week, to rate how negative each experience was on a scale of 0 (not at all) to 4 (extremely), and whether they believe each experience was caused by the app; (4) for participants randomized into the Spark arm, there were opportunities within the app that allowed for freeform text input. This freeform text was reviewed daily by study staff.

Data and incoming correspondence were reviewed daily and any identified potential clinical concerns were recorded by the study staff and verified by the study investigators. All logged clinical concerns were reviewed daily by a study investigator who determined whether the clinical concern required escalation to the study clinician (Dr Raph Rose), an independent licensed clinical psychologist not otherwise associated with the study or study sponsor, for clinical input or follow-up with the participant. The study clinician would then determine whether the participant was safe and eligible to continue with the study based on the information provided or based on contact with the participant or legal guardian. If a clinical concern related to suicidality was endorsed during the onboarding session, a study investigator administered the Ask Suicide-Screening Questions Toolkit [64] to the participant to determine the imminent risk level, recommend emergency resources if required, and escalate to the study clinician for follow-up.

Participants were withdrawn from the study if clinical concerns met the following criteria: (1) if the study clinician determined that the participant was no longer eligible to continue with the study, (2) if the clinician could not monitor safety because of not being able to reach the participant or other listed contacts, and (3) if the clinician could not monitor safety because participants did not complete the weekly symptom check questionnaire for 2 consecutive weeks. In any of these circumstances, the participant was informed, withdrawn from the study, and sent a list of mental health resources via email.

### Safety Classification

Safety classification was based on the following definitions set forth by the US Food and Drug Administration (FDA) and FDA-recognized consensus standards, *ISO 14155:2020 Clinical Investigation of Medical Devices for Human Subjects-Good Clinical Practice* [65-67]. After study completion, a clinician who was not otherwise involved in the study (JF) reviewed all clinical concern data and provided preliminary event classifications. Any events deemed to be potential adverse events (AEs) by this clinician were sent to the study clinician (Dr Rose) for external classification. Dr Rose was blinded to identifying participant information, participant ID, and JF’s ratings to make final determinations. Information was presented to Dr Rose in a manner that concealed the group condition. Nonetheless, it is possible that Dr Rose could discern the group identification for certain classifications based on the content provided by a participant. To reduce the potential for bias in classification, if Dr Rose provided a stricter classification rating than JF, that rating was maintained.

An AE was defined as an “untoward medical occurrence, unintended disease or injury, or untoward clinical signs (including abnormal laboratory findings) in subjects, users or other persons, whether or not related to the investigational medical device [67] and whether anticipated or unanticipated.”

An adverse device effect (ADE) was defined as an AE “related to the use of an investigational medical device” [67]. This includes any AE “resulting from insufficiencies or inadequacies in the instructions for use, the deployment, the implantation, the installation, the operation, or any malfunction of the investigational medical device.” This also includes “any event that is a result of a user error or intentional misuse” [67]. For this study, ADEs could have occurred in either the Spark or control arms.

A serious AE (SAE) or serious ADE was defined as an AE or ADE that met more than one of the following criteria: Resulted in fatality, posed a life-threatening risk or immediate risk of death at the time of occurrence, led to persistent or significant disability or incapacity, required prolonged inpatient hospitalization, represented an important medical event, as determined by appropriate medical judgment, that could jeopardize the participant’s well-being, or where medical or surgical intervention might be necessary to prevent one of the aforementioned outcomes. This did not include planned hospitalization for a preexisting condition [65].

Unanticipated ADEs (UADEs), as defined in the FDA regulation 21 Code of Federal Regulation 812.3 [65], also referred to as “unanticipated problems,” included any serious adverse effect on health or safety or any life-threatening problem or death caused by, or associated with, a device, if that effect, problem, or death was not previously identified in nature, severity, or degree of incidence in the investigational plan or application; or any other unanticipated serious problem associated with a device that relates to the rights, safety, or welfare of participants.

### Outcomes and Statistical Analysis

An a priori statistical analysis plan was restricted to participants with moderate to severe symptoms at baseline (PHQ-8≥10; moderate-to-severe cohort), unless otherwise specified. The α level was set to *P*=.05, and false discovery rate (FDR) correction for multiple comparisons was applied to the specified analyses.

### Depression Symptoms: PHQ-8

The primary outcome was a group difference in the change in depression symptoms from baseline to 5 weeks, as measured by the PHQ-8. A modified intention-to-treat (mITT) approach was used for primary analyses by including all participants randomized within the moderate-to-severe cohort. A per protocol (PP) analysis included data from participants in this cohort who had completed all weekly PHQ-8 questionnaires. Post hoc mITT and PP analyses were also conducted for participants with a baseline PHQ-8≥5 (mild-to-severe cohort) to evaluate the efficacy of Spark in participants with mild to severe symptoms of depression. Statistical analyses were performed using R version 4.1.2 (R Foundation for Statistical Computing) by an independent external statistician (LC) [68].

### Missing Data Analysis

Little’s test [69] was used to determine whether group differences existed in the proportion of missing PHQ-8 data across weeks for both moderate-to-severe and mild-to-severe cohorts. For any resulting significant results, the effects of known factors on missing data including Spark version, treatment group, week, baseline PHQ-8 severity, and age group were evaluated with follow-up *χ^2^* tests to determine whether data could be missing at random [70].

### Multiple Imputation Procedure

Multiple imputation was implemented on missing data from participants who had completed at least the baseline PHQ-8 assessment. Information on Spark version (2.1 and 2.2), treatment group, baseline PHQ-8 score, age group, week, and individual PHQ-8 item was included to impute 100 data sets with the Pan [71] method using the R *mice* package version 3.14.0 [68,72,73]. Missing PHQ-8 item–level scores and assessment completion days from baseline were imputed.

### Statistical Analysis

A linear mixed-effects model (LMM) analysis was implemented on an averaged imputed data set to evaluate the main effects of study arm (Spark and control) and week (0-5), and the study arm×week interaction using the R lme4 package (version 1.1.28 [74]). Four models were implemented in total, including the mITT and PP analyses for both the moderate-to-severe and mild-to-severe cohorts. In each model, group and week were entered as fixed factors. Spark version and assessment completion days from baseline were included as fixed factors to control for the effects of app version and differences in time between the completion of successive weekly assessments. PHQ-8 item–level scores and participants were included as random factors for the intercept, and the time from the baseline assessment was included as a random factor for the slope of participants. FDR correction was applied to the 4 *P* values for the study arm×week interaction effects. The effect size of the interaction (Cohen *f^2^*) was computed using pseudo *R^2^* (f*^2^*=R*^2^*/{1−R*^2^*}) [75,76] with the R MuMIn package (version 1.46.0 [77]). Dfs were estimated using the Satterthwaite method provided in the R lmerTest package (version 3.1-3) [78,79]. Follow-up analyses were performed to evaluate the effect of week within each group.

We also evaluated whether there was an average minimal clinically important difference (MCID) in symptom severity within each group, defined as a ≥5 point average decrease in PHQ-8 score between baseline and postintervention [80].

To assess the robustness of the study arm×week interaction, a generalized LMM (GLMM) with multiple imputation with the same model specification as the LMM analysis was implemented on item-level PHQ-8 data across the 100 imputed data sets using a 2-level Pan method [71], and the resulting 4 *P* values were FDR adjusted.

Group differences in remission rates, defined as PHQ-8 score <5 at postintervention [81], and treatment response rates, defined as a 50% reduction in PHQ-8 score between baseline and postintervention [50], were tested using *χ^2^* tests, and the resulting 4 *P* values were FDR adjusted.

### Secondary Clinical Outcomes

Secondary outcomes included anxiety symptoms (Generalized Anxiety Disorder Scale [59]), legal guardian–rated depression symptoms of their child (Mood and Feelings Questionnaire 61]), and participant-rated and legal guardian–rated (of their child) global functioning (Patient-Reported Outcomes Measurement Information System [60]) assessed at baseline and postintervention. Means, SDs, and 95% CIs for the average difference between baseline and postintervention were computed for these outcomes. A questionnaire was developed internally to assess the positive and negative effects of COVID-19, and the proportion of participants in each group that endorsed each item was computed.

### Safety

The total numbers of AE, ADE, SAEs, and UADE were computed per group for all randomized participants.

### Other Outcomes

Other outcomes measured included program adherence, engagement, and acceptability. Program adherence was assessed as the proportion of participants completing all sessions by postintervention and percent completion per module. Engagement for both study arms was assessed using the User Engagement Scale-short form [63], minutes spent in the app per week, and total app sessions. Usability was assessed using the system usability scale [62]. App acceptability was assessed with internally developed self-reported and legal guardian–reported ratings of the app with a 10-point Likert scale, asking how much the app improved mood or symptoms of depression and how enjoyable it was. Means, SDs, and mean differences between study arms with 95% CIs were calculated for these measures.

## Results

### Participants

**Figure 1:**
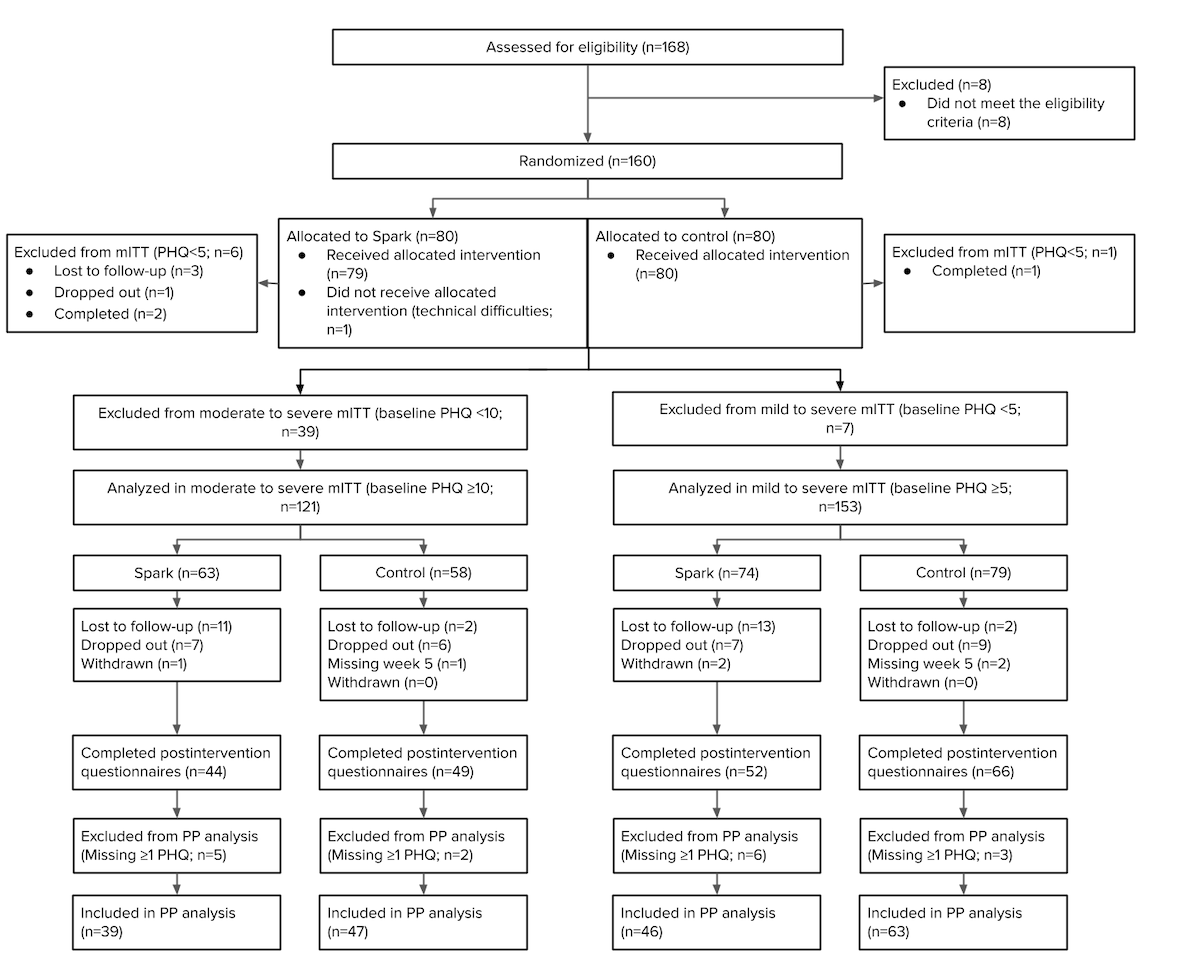
CONSORT (Consolidated Standards of Reporting Trials) diagram. The diagram excludes the control extension arm. Patients were considered lost to follow-up if they did not complete the postintervention questionnaire, considered to have dropped out if they missed 2 weekly safety checks, and considered to have withdrawn from the study if they asked to be removed. Patients were considered as missing week 5 if they did not complete the week 5 questionnaires but moved on to the control extension phase. mITT: modified intention-to-treat; PHQ-8: Patient Health Questionnaire-8; PP: per protocol.

A total of 168 adolescents consented to participate in this study between November 2020 and June 2021. The postintervention and follow-up data were collected between January 2021 and September 2021, when planned data collection had completed. Eight participants did not meet the inclusion criteria. A total of 160 patients were randomized (80 in the Spark arm; refer to Figure 1 for the CONSORT (Consolidated Standards of Reporting Trials) diagram; refer to Table 2 for the characteristics). In total, 41 participants received Spark version 2.1 and 39 participants received Spark version 2.2. Data from participants with a baseline PHQ-8 score ≥10 (moderate-to-severe cohort; Table 2) were included in all planned analyses, including mITT (n=121) and PP (n=86). Post hoc analyses included data from participants with a baseline PHQ-8 score≥5 (mild-to-severe cohort; Table 2) for mITT (n=153) and PP (n=109). Three participants skipped a question on the PHQ-8 assessment and were included in the PP analyses. The Spark and control arms did not differ significantly on any of the baseline demographic characteristics (all *P*>.05).

**Table 2:** Participant characteristics (N=160).

				Spark participants (n=80)		Control participants (n=80)
**Full sample, n (%)**				80 (100)		80 (100)
	Age (y), mean (SD)		16.89 (2.50)		16.79 (2.59)	
	**Gender, n (%)**					
		Female	50 (63)		51 (64)	
		Male	24 (30)		19 (24)	
		Nonbinary	6 (8)		10 (13)	
	**Race, n (%)**					
		American Indian or Alaska native	2 (3)		2 (3)	
		Asian	6 (8)		1 (1)	
		Black or African American	5 (6)		6 (8)	
		Pacific islander	0 (0)		0 (0)	
		Unknown	0 (0)		1 (1)	
		White	59 (74)		59 (74)	
		>1 race	8 (10)		11 (14)	
	**Ethnicity, n (%)**					
		Hispanic or Latinx	13 (16)		10 (13)	
		Not Hispanic or Latinx	61 (76)		64 (80)	
		Unknown	6 (8)		6 (8)	
	**Population density, n (%)**					
		Rural	2 (3)		2 (3)	
		Suburban	6 (8)		10 (13)	
		Urban	72 (90)		68 (85)	
	Depression diagnosis, n (%)		61 (76)		69 (86)	
	**Concurrent treatment, n (%)**					
		Psychotherapy	36 (34)		36 (34)	
		Medication	39 (37)		46 (43)	
		Other	4 (4)		5 (5)	
		None	26 (25)		19 (18)	
	**Past treatment, n (%)**					
		Psychotherapy	54 (44)		57 (44)	
		Medication	44 (36)		52 (40)	
		Other	11 (9)		9 (7)	
		None	15 (12)		11 (9)	
**Moderate-severe cohort (n=121), n (%)**				63 (100)		58 (100)
	Age (y), mean (SD)		17.06 (2.49)		16.46 (2.43)	
	**Gender, n (%)**					
		Female	42 (67)		39 (67)	
		Male	16 (25)		10 (17)	
		Nonbinary	5 (8)		9 (16)	
	**Race, n (%)**					
		American Indian or Alaska native	1 (2)		2 (3)	
		Asian	4 (6)		0 (0)	
		Black or African American	3 (5)		2 (3)	
		Pacific islander	0 (0)		0 (0)	
		Unknown	0 (0)		1 (2)	
		White	47 (75)		45 (78)	
		>1 race	8 (13)		8 (14)	
	**Ethnicity, n (%)**					
		Hispanic or Latinx	11 (18)		8 (14)	
		Not Hispanic or Latinx	46 (73)		47 (81)	
		Unknown	6 (10)		3 (5)	
	**Population density, n (%)**					
		Rural	1 (2)		1 (2)	
		Suburban	5 (8)		6 (10)	
		Urban	57 (90)		51 (88)	
	Depression diagnosis, n (%)		51 (81)		51 (88)	
	**Concurrent treatment, n (%)**					
		Psychotherapy	28 (34)		23 (31)	
		Medication	33 (40)		35 (47)	
		Other	4 (5)		3 (4)	
		None	18 (22)		14 (19)	
	**Past treatment, n (%)**					
		Psychotherapy	46 (46)		41 (43)	
		Medication	37 (37)		40 (42)	
		Other	10 (10)		6 (6)	
		None	8 (8)		8 (8)	
**Mild-to-severe cohort (n=153), n (%)**				74 (100)		79 (100)
	Age (y), mean (SD)		17.00 (2.48)		8 (13.8)	
	**Gender, n (%)**					
		Female	48 (65)		50 (63)	
		Male	20 (27)		19 (24)	
		Nonbinary	6 (8)		10 (13)	
	**Race, n (%)**					
		American Indian or Alaska native	2 (3)		2 (3)	
		Asian	4 (5)		1 (1)	
		Black or African American	4 (5)		5 (6)	
		Pacific islander	0 (0)		0 (0)	
		Unknown	0 (0)		1 (1)	
		White	56 (76)		59 (75)	
		>1 race	8 (11)		11 (14)	
	**Ethnicity, n (%)**					
		Hispanic or Latinx	13 (18)		10 (13)	
		Not Hispanic or Latinx	55 (74)		63 (80)	
		Unknown	6 (8)		6 (8)	
	**Population density, n (%)**					
		Rural	2 (3)		2 (3)	
		Suburban	6 (8)		10 (13)	
		Urban	67 (91)		67 (85)	
	Depression diagnosis, n (%)		58 (78)		68 (86)	
	**Concurrent treatment, n (%)**					
		Psychotherapy	33 (34)		35 (34)	
		Medication	38 (38)		45 (43)	
		Other	4 (4)		5 (5)	
		None	23 (24)		19 (18)	
	**Past treatment, n (%)**					
		Psychotherapy	51 (44)		56 (44)	
		Medication	43 (37)		51 (40)	
		Other	11 (9)		9 (7)	
		None	12 (10)		11 (9)	

### Treatment Outcomes: PHQ-8

Weekly means, SD, and 95% CIs for baseline to postintervention change scores per group; remission and treatment response rates per group are reported in Table 3.

**Table 3:** Patient Health Questionnaire-8 descriptives.

Cohort and group			Baseline score, mean (SD)		Week 1 score, mean (SD)		Week 2 score, mean (SD)		Week 3 score, mean (SD)		Week 4 score, mean (SD)		Postintervention score, mean (SD)		Postbaseline score, mean difference (95% CI)		Remission, n (%)		Treatment response, n (%)
**Moderate to severe (n=121)**																			
	Spark (n=63)	15.78 (3.62)		13.04 (5.06)		11.70 (5.56)		11.80 (5.55)		10.75 (6.13)		10.70 (5.54)		−5.08 (−6.72 to −3.42)		11 (17)		15 (24)	
	Control (n=58)	15.31 (3.42)		13.06 (4.37)		12.30 (4.77)		11.68 (4.88)		11.70 (5.69)		11.80 (4.99)		−3.51 (−5.09 to −1.93)		2 (3)		8 (14)	
**Mild to severe (n=153)**																			
	Spark (n=74)	14.36 (4.78)		11.99 (5.39)		10.73 (5.70)		10.65 (5.88)		9.56 (6.38)		9.51 (5.91)		−4.85 (−6.59 to −3.09)		20 (27)		22 (30)	
	Control (n=79)	13.29 (4.51)		11.59 (4.73)		10.88 (5.09)		10.25 (5.09)		10.56 (5.47)		10.43 (5.39)		−2.86 (−4.43 to −1.30)		9 (11)		12 (15)	

### Moderate-to-Severe Cohort

#### Modified Intention to Treat (n=121)

**Figure 2:**
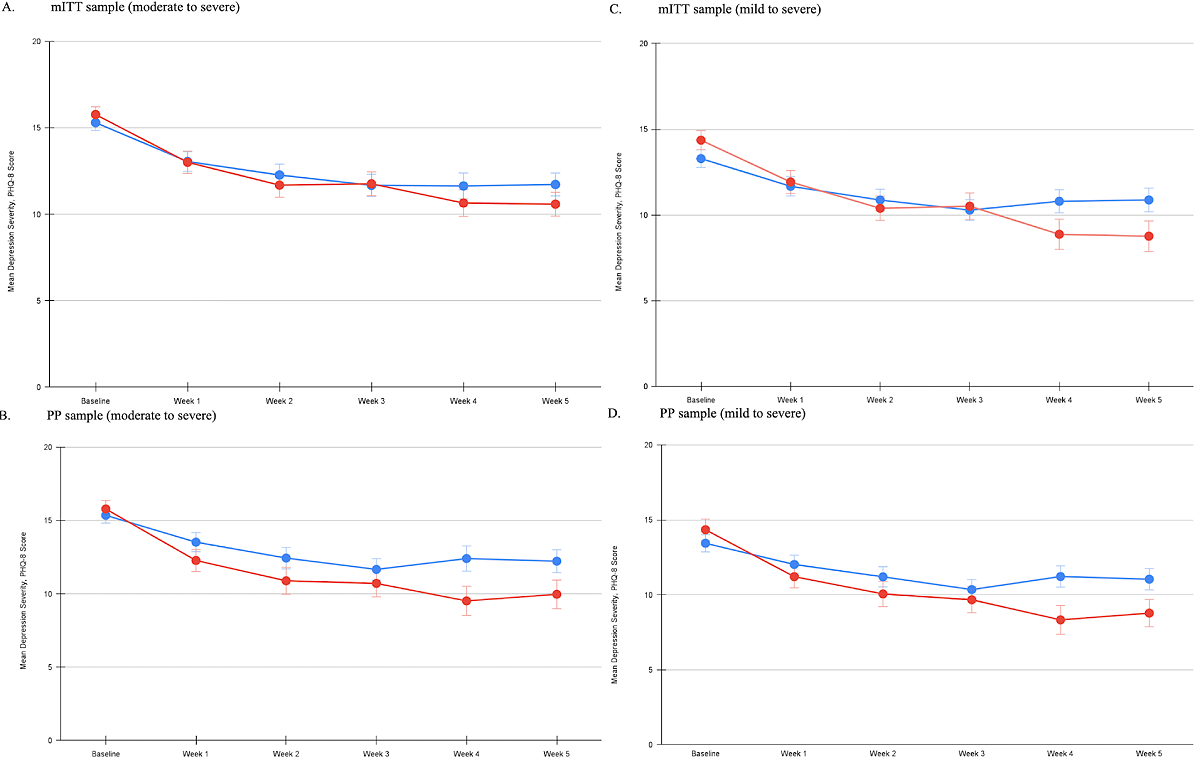
Depression severity by week: (A) modified intention-to-treat (mITT) moderate to severe cohort, (B) per protocol (PP) moderate to severe cohort, (C) mITT mild to moderate cohort, and (D) PP mild to moderate cohort. Note that the graphs depict average Patient Health Questionnaire-8 (PHQ-8) scores at each time point based on observed data.

The LMM revealed a nonsignificant study arm x week interaction (*t*_127.66_=−1.911; *P*=.06; FDR adjusted *P*=.06; f^2^=0.0012; power: 0.468-0.494; Figure 2A). There was a nonsignificant effect of study arm (*t*_177.54_=.573; *P*=.57; f^2^=0.00034; power: 0.069-0.079) and a significant effect of week (*t*_1977.06_=−4.395; *P*<.001; f^2^=0.00215; power: 0.985-0.993). There was a significant effect of week in the Spark arm (*t*_1759.08_=−3.508; *P*<.001; change score=−5.08, 95% CI −6.72 to −3.42) and control arm (*t*_561.98_=−3.244; *P*=.001; change score=−3.51, 95% CI −5.09 to −1.93). The GLMM also produced a nonsignificant study arm x week interaction (*F*_1,24186.41_=3.223; *P*=.07; FDR adjusted *P*=.07).

At the end of the intervention period, the Spark arm showed significantly higher remission rates compared with the control arm (Spark, 11/63, 17% and control, 2/58, 3%; *χ^2^*_1_=6.183; *P*=.01; FDR adjusted *P*=.03). Treatment response rates between the study arms (Spark, 15/63, 24% and control, 8/58, 14%) were not statistically significant (*χ^2^*_1_=1.968; *P*=.07; FDR adjusted *P*=.16).

#### Per Protocol (n=86)

The LMM revealed a significant study arm x week interaction (*t*_91.59_=−2.546; *P*=.01; FDR adjusted *P*=.02; f^2^=0.0107; power: 0.699-0.699; Figure 2B). There was a nonsignificant effect of study arm (*t*_82.43_=−0.072; *P*=.94; f^2^=.00031; power: 0.041-0.056) and a significant effect of week (*t*_1511_=−4.876; *P*<.001; f^2^=0.00425; power: 0.995-0.997). There was a significant effect of week in the Spark arm (*t*_1183.54_=−4.242; *P*<.001; change score=−5.82, 95% CI −7.54 to −4.10) and the control arm (*t*_532.3_=−3.482; *P*<.001; change score=−3.13, 95% CI −4.49 to −1.76). The GLMM also produced a significant study arm x week interaction (*F*_1,6.569e+08_=6.624; *P*=.01; FDR adjusted *P*=.01).

### Mild-Severe Cohort

#### Modified Intention to Treat (n=153)

The LMM revealed a significant study arm x week interaction (*t*_160.20_=−3.054; *P*=.003; FDR adjusted *P*=.005; f^2^=0.00036; power: 0.846-0.884; Figure 2C). There was a nonsignificant effect of study arm (*t*_149.68_=1.272; *P*=.21; f^2^=.00157; power: 0.233-0.275) and a significant effect of week (*t*_2539.94_=−3.06; *P*=.002; f^2^=0.00058; power: 0.861-0.868). There was a significant effect of week in the Spark arm (*t*_1697.40_=−3.32; *P*<.001; change score=−4.85, 95% CI −6.59 to −3.09) but not in the control arm (*t*_1177.40_=−1.838; *P*=.07; change score=−2.86, 95% CI −4.43 to −1.30). The GLMM also produced a significant study arm x week interaction (*F*_1,19992.155_=8.21; *P*=.004; FDR adjusted *P*=.008).

At the end of the intervention period, the Spark arm showed significantly higher remission (Spark, 20/74, 27% and control, 9/79, 11%; *χ^2^*_1_=6.1; *P*=.01; FDR adjusted *P*=.03) and treatment response (Spark, 22/74, 30% and control,12/79, 15%; *χ^2^*_1_=4.7; *P*=.03; FDR adjusted *P*=.04) rates compared with the control arm.

#### Per Protocol (n=109)

The LMM revealed a significant study arm x week interaction (*t*_115.09_=−3.603; *P*<.001; FDR adjusted *P*=.002; f^2^=0.009498; power: 0.941-0.949; Figure 2D). There was a nonsignificant effect of study arm (*t*_105.69_=0.544; *P*=.59; f^2^=.00006; power: 0.078-0.086) and a significant effect of week (*t*_1955.64_=−3.6; *P*<.001; f^2^=0.00169; power: 0.936~0.939). There was a significant effect of week in the Spark arm (*t*_1178.37_=−4.049; *P*<.001; change score=−5.57, 95% CI −7.04 to −4.09) and the control arm (*t*_1011.58_=−2.231; *P*=.03; change scores=−2.39, 95% CI −3.60 to −1.19). The GLMM also produced a significant study arm x week interaction (*F*_1,1.026e+09_=13.205; *P*<.001; FDR adjusted *P*<.001).

Notably, all analyses that showed an uncorrected α threshold of *P*<.05 remained significant following FDR correction, and study arm×week interaction effects were consistent across LMM and GLMM analyses.

Little’s test was significant for moderate-to-severe (*χ^2^*_34_=72.5; *P*<.001) and mild-to-severe cohorts (*χ^2^*_39_=106.94; *P*<.001), suggesting that data were not missing completely at random. Missing data patterns were associated with observed variables such as treatment group, week, and age group (Multimedia Appendix 1), suggesting that data are missing at random. No analysis showed a significant effect of Spark app version (significance *P*>.05).

### Secondary Clinical Outcomes

Means, SDs, and 95% CIs of the difference between baseline and postintervention scores for all secondary participant-reported and legal guardian–reported measures are reported for each group in Table 4. The results for the effects of COVID-19 in the moderate-to-severe cohort are presented in Table 5. Notably, 49.2% (59/120) of the participants reported reduced access to mental health resources during the pandemic at baseline.

**Table 4:** Secondary clinical measures: participant and legal guardian (moderate-to-severe cohort; n=121).

			Spark participant scores							Control participant scores				
			Baseline, mean (SD)		Post, mean (SD)		Postbaseline, mean difference (95% CI)		Baseline, mean (SD)			Post, mean (SD)		Postbaseline, mean difference (95% CI)
**Participant measures: Spark (n=43) and control (n=49)**														
	GAD-7^a^	12.26 (4.94)		9.05 (5.57)		−3.21 (−4.61 to −1.81)		12.31 (4.54)			10.86 (4.83)		−1.45 (−2.41 to −0.49)	
	PROMIS^b^ 7+2– General Health	19.51 (3.13)		20.86 (4.01)		1.35 (0.30 to 2.40)		19.61 (3.34)			19.27 (3.64)		−0.35 (−0.88 to 0.19)	
	PROMIS 7+2–Fatigue	4.35 (0.90)		4.00 (1.11)		−0.35 (−0.66 to −0.03)		4.39 (0.84)			4.29 (0.91)		−0.10 (−0.34 to 0.13)	
	PROMIS 7+2–Pain	3.00 (1.21)		2.72 (1.39)		−0.28 (−0.74 to 0.18)		2.90 (1.29)			2.98 (1.31)		0.08 (−0.34 to 0.50)	
	BRS^c^	2.56 (0.66)		—^d^		—		2.52 (0.73)			—		—	
**Legal guardian measures: Spark (n=24) and control (n=28)**														
	MFQ-SF^e,f^	13.61 (4.71)		8.78 (4.99)		−4.83 (−6.69 to −2.96)		12.89 (5.62)			10.04 (4.43)		−2.85 (−4.49 to −1.21)	
	PROMIS 7+2 Parent Proxy–General Health	21.50 (2.28)		22.71 (3.93)		1.21 (−0.16 to 2.57)		21.04 (3.89)			22.07 (3.92)		1.04 (0.08 to 1.99)	
	PROMIS 7+2 Parent Proxy–Fatigue	3.96 (1.00)		3.63 (0.97)		−0.33 (−0.79 to 0.13)		3.89 (0.92)			3.21 (0.99)		−0.69 (−1.10 to −0.26)	
	PROMIS 7+2 Parent Proxy–Pain	2.96 (1.00)		2.88 (0.99)		−0.08 (−0.67 to 0.50)		2.61 (1.13)			2.39 (1.20)		−0.21 (−0.61 to 0.19)	

^a^GAD-7: Generalized Anxiety Disorder-7.

^b^PROMIS: Patient-Reported Outcomes Measurement Information System.

^c^BRS: Brief Resilience Scale.

^d^Not available; data were not collected.

^e^MFQ-SF: Mood and Feelings Questionnaire-Short Form.

^f^A total of 2 legal guardians (1 in the Spark arm and 1 in the control arm) completed the PROMIS but not the Mood and Feelings Questionnaire.

**Table 5:** Positive and negative impacts of COVID-19—participant and legal guardian (moderate-to-severe cohort).

			Baseline: Spark, n (%)	Baseline: control, n (%)	Post: Spark, n (%)	Post: control, n (%)
**Participant (moderate-to-severe cohort)**						
	**Negative impacts of COVID-19**					
		Difficulty with schoolwork	49/62 (79)	48/58 (83)	29/43 (67)	36/49 (74)
		Job loss or worry about employment for you or someone you care about	21/62 (34)	15/58 (26)	18/43 (42)	15/49 (31)
		Loss of family member or someone you care about	8/62 (12)	10/58 (17)	5/43 (12)	8/49 (16)
		Missed milestones (eg, prom or graduation) or meaningful holiday celebrations	45/62 (73)	36/58 (62)	28/43 (65)	31/49 (63)
		None	1/62 (2)	1/58 (2)	3/43 (7)	0/49 (0)
		Other	3/62 (5)	3/58 (55)	1/43 (25)	2/49 (45)
		Personal illness	7/62 (115)	11/58 (19)	7/43 (16)	6/49 (12)
		Reduced access to mental health resources or support	33/62 (53)	26/58 (45)	17/43 (40)	23/49 (47)
		Serious illness of family member or someone you care about	10/62 (16)	10/58 (17)	11/43 (26)	10/49 (20)
		Social isolation/loneliness	58/62 (94)	51/58 (88)	36/43 (84)	44/49 (90)
		Worry about having enough food	6/62 (10)	5/58 (9)	5/43 (12)	4/49 (8)
		Worry about housing	6/62 (10)	3/58 (5)	4/43 (9)	8/49 (16)
		Worry about personal or family finances	27/62 (44)	23/58 (40)	19/43 (44)	25/49 (51)
		Worry about physical violence in the home or local community	6/62 (10)	4/58 (7)	3/43 (7)	8/49 (16)
	**Positive impacts of COVID-19**					
		Increased in-person time with family or household members	21/63 (33)	26/58 (45)	22/44 (50)	24/49 (49)
		Increased time for self-care (eg, exercise)	22/63 (35)	24/58 (41)	26/44 (59)	24/49 (49)
		Increased virtual time with family or friends	13/63 (21)	13/58 (22)	12/44 (27)	9/49 (18)
		None	18/63 (29)	12/58 (21)	5/44 (11)	8/49 (16)
		Other	1/68 (2)	3/58 (5)	1/44 (2)	0/49 (0)
		Reduced academic stress	8/63 (13)	9/58 (16)	12/44 (27)	13/49 (27)
		Reduced social stress	23/63 (37)	20/58 (34)	22/44 (50)	17/49 (35)
**Legal guardian (moderate-to-severe cohort)**						
	**Negative impacts of COVID-19**					
		Difficulty with schoolwork	23/33 (70)	24/35 (69)	16/24 (67)	19/28 (68)
		Job loss or worry about employment for your child or someone your child cares about	3/33 (9)	3/35 (9)	3/24 (13)	3/28 (111)
		Loss of family member or someone your child cares about	3/33 (9)	2/35 (6)	2/24 (8)	2/28 (7)
		Missed milestones (eg, prom or graduation) or meaningful holiday celebrations	18/33 (55)	23/35 (66)	7/24 (29)	13/28 (46)
		None	0/33 (0)	0/35 (0)	3/24 (13)	1/28 (4)
		Other	6/33 (18)	8/35 (23)	2/24 (8)	1/28 (4)
		Personal illness	2/33 (6)	4/35 (11)	2/24 (8)	2/28 (7)
		Reduced access to mental health resources or support	14/33 (42)	16/35 (46)	9/24 (38)	10/28 (36)
		Serious illness of family member or someone your child cares about	4/33 (12)	6/35 (17)	4/24 (17)	2/28 (7)
		Social isolation, loneliness, or both	33/33 (100)	35/35 (100)	20/24 (83)	24/28 (86)
		Worry about having enough food	2/33 (6)	1/35 (3)	0/24 (0)	2/28 (7)
		Worry about housing	1/33 (3)	1/35 (3)	1/24 (4)	2/28 (7)
		Worry about personal or family finances	8/33 (24)	14/35 (40)	6/24 (25)	10/28 (36)
		Worry about physical violence in the home or local community	2/33 (6)	2/35 (6)	1/24 (4)	3/28 (11)
	**Positive impacts of COVID-19**					
		Increased in-person time with family or household members	24/31 (77)	22/35 (63)	16/24 (67)	18/28 (64)
		Increased time for self-care (eg, exercise)	9/31 (29)	13/35 (37)	9/24 (38)	6/28 (21)
		Increased internet-based time with family or friends	6/31 (19)	14/35 (40)	6/24 (25)	6/38 (21)
		None	2/31 (6)	5/35 (14)	6/24 (25)	5/28 (18)
		Other	1/31 (3)	1/35 (3)	0/24 (0)	1/28 (4)
		Reduced academic stress	8/31 (26)	6/35 (17)	7/24 (29)	8/28 (29)
		Reduced social stress	9/31 (29)	15/35 (43)	4/24 (17)	8/28 (29)

### Safety Outcomes

There were no SAEs, ADEs, or UADEs in either group. There were a total of 2 AEs in the control arm and 4 in the Spark arm. Both AEs in the control arm were clinical deteriorations in PHQ-8 scores for 2 consecutive weeks. In the Spark arm, there was 1 clinical deterioration in the PHQ-8 score for 2 consecutive weeks, 2 instances of participant-reported suicidal ideation, and 1 instance of a legal guardian reporting that the participant was experiencing nonsuicidal self-injury ideation.

### Adherence, Acceptability, and Engagement

In the Spark arm, 87% (55/63) of participants completed module 1, 79% (50/63) completed module 2, 64% (40/64) completed module 3, 48% (30/63) completed module 4, and 40% (25/63) completed module 5. App acceptability metrics are reported in Table 6. Engagement with the app as defined as the average number of sessions are reported in Table 7.

**Table 6:** Spark and control app acceptability ratings: participant and legal guardian (moderate-to-severe cohort)

	Participant ratings			Legal guardian ratings		
	Spark (n=44), mean (SD)	Control (n=49), mean (SD)	Mean difference (95% CI)	Spark (n=28), mean (SD)	Control (n=24), mean (SD)	Mean difference (95% CI)
Mood or depression symptom improvement	5.20 (2.04)	2.51 (2.41)	2.69 (1.76 to 3.62)	4.00 (2.17)	3.75 (2.47)	0.25 (−1.04 to 1.54)
Enjoyable	7.75 (2.01)	5.24 (2.72)	2.51 (1.52 to 3.50)	5.63 (2.18)	4.86 (2.09)	0.77 (−0.43 to.197)
SUS^a^	82.85 (12.42)	76.12 (13.47)	6.73 (1.02 to 11.74)	N/A^b^	N/A	N/A
UES-SF^c^	3.81 (0.53)	3.22 (0.65)	0.59 (0.34 to 0.84)	N/A	N/A	N/A
UES-SF: focused attention	2.77 (0.89)	2.29 (0.78)	0.48 (0.14 to 0.82)	N/A	N/A	N/A
UES-SF: perceived usability	4.19 (0.77)	4.14 (0.89)	0.05 (−0.29 to.39)	N/A	N/A	N/A
UES-SF: esthetic appeal	4.30 (0.63)	3.03 (0.89)	1.27 (0.95 to 1.59)	N/A	N/A	N/A
UES-SF: reward	3.99 (0.84)	3.43 (0.95)	0.56 (0.19 to 0.93)	N/A	N/A	N/A

^a^SUS: systems usability scale.

^b^N/A: not applicable.

^c^UES-SF: User Engagement Scale-short form.

**Table 7:** Weekly Spark and control number of app sessions.

	Spark participant app sessions, mean (SD)	Control participant app sessions, mean (SD)
Week 1	7.57 (4.71)	3.53 (2.22)
Week 2	4.78 (3.84)	2.64 (1.62)
Week 3	4.19 (2.97)	2.40 (1.50)
Week 4	3.60 (2.94)	2.40 (1.63)
Week 5	3.46 (3.42)	2.33 (1.82)
Total	23.60 (13.75)	13.29 (6.74)

## Discussion

### Principal Findings

This RCT showed that, in planned and exploratory analyses, Spark was efficacious in treating mild to severe symptoms of depression, including a clinically meaningful reduction in depression symptoms and a significantly larger reduction in depression symptoms as well as higher remission and treatment response rates at postintervention compared with an active control. Spark did not negatively impact participant safety, as demonstrated by having no ADEs reported A mITT analysis, restricted to participants with moderate to severe symptoms at baseline, showed no group differences in symptom change from baseline to postintervention. The treatment response and remission rates for the Spark arm were consistent with those found in other digital CBT apps [50], and the Spark arm showed significantly higher remission rates compared with the control arm. A follow-up PP analysis restricted to participants who consistently engaged with their assigned app per the study protocol revealed that the Spark arm showed a significantly larger decrease in depression symptoms compared with the control arm. This finding supports the idea that engagement and adherence are important drivers of treatment outcomes [82,83]. Indeed, engagement with and adherence to Spark were also high, compared with other digital therapeutic studies, and higher than most popular wellness apps [82-86]. Participants also rated Spark as moderately enjoyable, usable, and successful in improving mood and depression symptoms [87]. Although out of scope for this study, future research should directly investigate engagement in clinical outcomes, especially in the context of specific therapeutic components of the intervention, such as mood logs and BAs, and how they may mediate symptom change.

Notably, exploratory analyses including a larger cohort of participants with mild to severe symptoms at baseline showed significant group differences in symptom reduction, remission rate, and treatment response rate. The Spark arm within this cohort also showed an MCID within a 95% CI. Statistical tests were robust to correction for multiple comparisons and suggest that Spark may be an effective treatment for a wide range of depression severity, including those with mild symptoms, consistent with a recent meta-analysis of digital CBT [25]. As mild symptoms are often overlooked and, when untreated, can worsen or have long-term consequences [88], self-guided DTx may be a particularly attractive treatment option for this severity group.

The results also suggest that Spark may be promising as an adjunct treatment and part of a stepped care model for depression treatment in adolescents [89]. Participants were able to continue their usual care while enrolled in the study, and more than three quarters (130/160, 81.3%) of the participants reported having been diagnosed with depression and about half were receiving some form of concurrent treatment for depression (medication, 85/160, 53.1% or psychotherapy, 72/160, 45%). Adjunct digital treatments can reduce the time and cost of standard care [84,90], and as a self-contained treatment option, Spark can be easily integrated into a wide range of treatment plans.

These results are also promising in light of issues with access to mental health care for adolescents, many of which were magnified during the COVID-19 pandemic along with an increase in prevalence rates [1,17-19,21]. Approximately 48.8% (59/121) of the participants identified reduced access to mental health resources during the pandemic, underscoring the treatment potential of self-guided evidence-based DTx, such as Spark, which can be made available immediately, widely, and equitably, when other forms of care are limited.

### Limitations

The study was not statistically powered to detect group differences in PHQ-8 symptom change; therefore, these results should be replicated in a fully powered sample. Powered analyses are also warranted to directly investigate the impacts of engagement, baseline symptom severity, and concurrent treatments on outcomes. Although an active control was used, the open-label design of the study is a limitation, as participants were not blinded to treatment assignment and the primary outcome was a self-report PHQ-8; blinded studies with clinician-reported outcomes will provide stronger evidence of efficacy. The exploratory results including adolescents with mild to severe symptoms at baseline, although promising, should be interpreted with caution and replicated. Similarly, although our PP analysis indicates that engagement with Spark may drive symptom improvement, these observations may be owing to survivorship bias. The eligibility criteria for this study were intentionally designed to allow adolescents who were seeking resources during the pandemic to participate and did not require stability on concurrent treatment, an assessment for treatment resistance, or stratification of study arms based on concurrent treatment, which may have introduced noise into the data.

### Generalizability

Spark was designed as a brief (5 weeks), adjunct to standard of care intervention to reduce symptoms of depression in adolescents (aged 13-21 years as defined by the FDA for medical devices). As such, the results should be interpreted for an adolescent sample; we would not anticipate these results to generalize to the broader adult and child population. We recruited participants directly (ie, through social media advertising rather than through a health care provider), which could have created a self-selection bias. To evaluate the generalizability of these results in a naturalistic setting, future studies should evaluate the efficacy of Spark as a treatment for depression in patients who are introduced to Spark by a health care provider. Future studies should evaluate long-term outcomes and future product developments (eg, evergreen features).

### Conclusions

DTx such as Spark could help overcome known barriers to access to care [14,16,91,92] by providing an immediate treatment option and serve as an effective adjunct form of care. DTx can also serve to reduce stigma around mental health treatment, as interventions can be delivered privately, and can improve treatment adherence by providing an engaging experience. This study provides preliminary evidence that a brief, self-guided, CBT-based DTx can be used to effectively and safely treat mild to severe depression symptoms in adolescents.

## Appendix

Multimedia Appendix 1 Missing data analysis by cohort.

Multimedia Appendix 2 CONSORT-eHEALTH checklist (V 1.6.1).

## Abbreviations

ADE: adverse device effect
AE: adverse event
BA: behavioral activation
CBT: cognitive behavioral therapy
CONSORT: Consolidated Standards of Reporting Trials
DTx: digital therapeutics
FDA: US Food and Drug Administration
FDR: false discovery rate
GLMM: generalized linear mixed-effects model
LMM: linear mixed-effects model
MCID: minimal clinically important difference
mITT: modified intention-to-treat
PHQ-8: Patient Health Questionnaire-8
PP: per protocol
RCT: randomized controlled trial
SAE: serious adverse event
UADE: unanticipated adverse device effect

## References

[1] Data and statistics on children's mental health. Centers for Disease Control and Prevention.

[2] Mojtabai R, Olfson M, Han B (2016). National trends in the prevalence and treatment of depression in adolescents and young adults. Pediatrics.

[3] Keyes KM, Gary D, O'Malley PM, Hamilton A, Schulenberg J (2019). Recent increases in depressive symptoms among US adolescents: trends from 1991 to 2018. Soc Psychiatry Psychiatr Epidemiol.

[4] Mental illness. National Institute of Mental Health (NIMH).

[5] Zisook S, Lesser I, Stewart JW, Wisniewski SR, Balasubramani GK, Fava M, Gilmer WS, Dresselhaus TR, Thase ME, Nierenberg AA, Trivedi MH, Rush AJ (2007). Effect of age at onset on the course of major depressive disorder. Am J Psychiatry.

[6] Alegría M, Green JG, Mclaughlin KA, Loder S (2015). Disparities in child and adolescent mental health and mental health services in the U.S. William T. Grant Foundation.

[7] Das JK, Salam RA, Lassi ZS, Khan MN, Mahmood W, Patel V, Bhutta ZA (2016). Interventions for adolescent mental health: an overview of systematic reviews. J Adolesc Health.

[8] Edgcomb JB, Zima B (2018). Medication adherence among children and adolescents with severe mental illness: a systematic review and meta-analysis. J Child Adolesc Psychopharmacol.

[9] Gearing RE, Schwalbe CS, Short KD (2012). Adolescent adherence to psychosocial treatment: mental health clinicians' perspectives on barriers and promoters. Psychother Res.

[10] Rickwood D, Deane FP, Wilson CJ, Ciarrochi J (2005). Young people's help-seeking for mental health problems. Adv Ment Health.

[11] Mehra K, Hawke LD, Watson P, Sheikhan NY, Leroux E, Henderson J (2021). Youth perspectives on seeking psychotherapy: a concurrent mixed methods study. J Can Acad Child Adolesc Psychiatry.

[12] Westin AM, Barksdale CL, Stephan SH (2014). The effect of waiting time on youth engagement to evidence based treatments. Community Ment Health J.

[13] Bodden DH, Stikkelbroek Y, Dirksen CD (2018). Societal burden of adolescent depression, an overview and cost-of-illness study. J Affect Disord.

[14] Sheppard R, Deane FP, Ciarrochi J (2018). Unmet need for professional mental health care among adolescents with high psychological distress. Aust N Z J Psychiatry.

[15] El-Amin T, Anderson BL, Leider JP, Satorius J, Knudson A (2018). Enhancing mental health literacy in rural America: growth of mental health first aid program in rural communities in the united states from 2008–2016. J Rural Ment Health.

[16] Cunningham PJ (2009). Beyond parity: primary care physicians' perspectives on access to mental health care. Health Aff (Millwood).

[17] Murthy VH (2021). Protecting youth mental health: the U.S. surgeon general’s advisory. US Department of Health and Human Services.

[18] Racine N, Cooke JE, Eirich R, Korczak DJ, McArthur B, Madigan S (2020). Child and adolescent mental illness during COVID-19: a rapid review. Psychiatry Res.

[19] Zhang L, Zhang D, Fang J, Wan Y, Tao F, Sun Y (2020). Assessment of mental health of Chinese primary school students before and after school closing and opening during the COVID-19 pandemic. JAMA Netw Open.

[20] Stinson EA, Sullivan RM, Peteet BJ, Tapert SF, Baker FC, Breslin FJ, Dick AS, Gonzalez MR, Guillaume M, Marshall AT, McCabe CJ, Pelham 3rd WE, Van Rinsveld A, Sheth CS, Sowell ER, Wade NE, Wallace AL, Lisdahl KM (2021). Longitudinal impact of childhood adversity on early adolescent mental health during the COVID-19 pandemic in the ABCD study cohort: does race or ethnicity moderate findings?. Biol Psychiatry Glob Open Sci.

[21] Panchal U, Salazar de Pablo G, Franco M, Moreno C, Parellada M, Arango C, Fusar-Poli P (2023). The impact of COVID-19 lockdown on child and adolescent mental health: systematic review. Eur Child Adolesc Psychiatry.

[22] Vigerland S, Lenhard F, Bonnert M, Lalouni M, Hedman E, Ahlen J, Olén O, Serlachius E, Ljótsson B (2016). Internet-delivered cognitive behavior therapy for children and adolescents: a systematic review and meta-analysis. Clin Psychol Rev.

[23] Fleming T, Bavin L, Lucassen M, Stasiak K, Hopkins S, Merry S (2018). Beyond the trial: systematic review of real-world uptake and engagement with digital self-help interventions for depression, low mood, or anxiety. J Med Internet Res.

[24] Martinengo L, Stona AC, Griva K, Dazzan P, Pariante CM, von Wangenheim F, Car J (2021). Self-guided cognitive behavioral therapy apps for depression: systematic assessment of features, functionality, and congruence with evidence. J Med Internet Res.

[25] Karyotaki E, Efthimiou O, Miguel C, Bermpohl FM, Furukawa TA, Cuijpers P, Riper H, Patel V, Mira A, Gemmil AW, Yeung AS, Lange A, Williams AD, Mackinnon A, Geraedts A, van Straten A, Meyer B, Björkelund C, Knaevelsrud C, Beevers CG, Botella C, Strunk DR, Mohr DC, Ebert DD, Kessler D, Richards D, Littlewood E, Forsell E, Feng F, Wang F, Andersson G, Hadjistavropoulos H, Christensen H, Ezawa ID, Choi I, Rosso IM, Klein JP, Shumake J, Garcia-Campayo J, Milgrom J, Smith J, Montero-Marin J, Newby JM, Bretón-López J, Schneider J, Vernmark K, Bücker L, Sheeber LB, Warmerdam L, Farrer L, Heinrich M, Huibers MJ, Kivi M, Kraepelien M, Forand NR, Pugh N, Lindefors N, Lintvedt O, Zagorscak P, Carlbring P, Phillips R, Johansson R, Kessler RC, Brabyn S, Perini S, Rauch SL, Gilbody S, Moritz S, Berger T, Pop V, Kaldo V, Spek V, Forsell Y, Individual Patient Data Meta-Analyses for Depression (IPDMA-DE) Collaboration (2021). Internet-based cognitive behavioral therapy for depression: a systematic review and individual patient data network meta-analysis. JAMA Psychiatry.

[26] Goldberg SB, Lam SU, Simonsson O, Torous J, Sun S (2022). Mobile phone-based interventions for mental health: a systematic meta-review of 14 meta-analyses of randomized controlled trials. PLOS Digit Health.

[27] Leech T, Dorstyn D, Taylor A, Li W (2021). Mental health apps for adolescents and young adults: a systematic review of randomised controlled trials. Child Youth Serv Rev.

[28] Gräfe V, Greiner W (2017). Internet based treatment of depressive symptoms – a health economic evaluation of costs and benefits. Value Health.

[29] Hedman E, Ljótsson B, Lindefors N (2012). Cognitive behavior therapy via the Internet: a systematic review of applications, clinical efficacy and cost-effectiveness. Expert Rev Pharmacoecon Outcomes Res.

[30] DTA DTx definition and core principles. Digital Therapeutics Alliance.

[31] Topooco N, Berg M, Johansson S, Liljethörn L, Radvogin E, Vlaescu G, Nordgren LB, Zetterqvist M, Andersson G (2018). Chat- and internet-based cognitive-behavioural therapy in treatment of adolescent depression: randomised controlled trial. BJPsych Open.

[32] Wannachaiyakul S, Thapinta D, Sethabouppha H, Thungjaroenkul P, Likhitsathian S (2017). Randomized controlled trial of computerized cognitive behavioral therapy program for adolescent offenders with depression. Pac Rim Int J Nurs Res Thail.

[33] MacDonell KW, Prinz RJ (2017). A review of technology-based youth and family-focused interventions. Clin Child Fam Psychol Rev.

[34] Keles S, Bringedal G, Idsoe T (2021). Assessing fidelity to and satisfaction with the “Adolescent Coping with Depression Course” (ACDC) intervention in a randomized controlled trial. J Ration Emot Cogn Behav Ther.

[35] Brezing CA, Brixner DI (2022). The rise of prescription digital therapeutics in behavioral health. Adv Ther.

[36] Thase ME, McCrone P, Barrett MS, Eells TD, Wisniewski SR, Balasubramani GK, Brown GK, Wright JH (2020). Improving cost-effectiveness and access to cognitive behavior therapy for depression: providing remote-ready, computer-assisted psychotherapy in times of crisis and beyond. Psychother Psychosom.

[37] Oh S, Choi J, Han DH, Kim E (2023). Effects of game-based digital therapeutics on attention deficit hyperactivity disorder in children and adolescents as assessed by parents or teachers: a systematic review and meta-analysis. Eur Child Adolesc Psychiatry (Forthcoming).

[38] Hollis C, Falconer CJ, Martin JL, Whittington C, Stockton S, Glazebrook C, Davies EB (2017). Annual research review: digital health interventions for children and young people with mental health problems - a systematic and meta-review. J Child Psychol Psychiatry.

[39] Kollins SH, Childress A, Heusser AC, Lutz J (2021). Effectiveness of a digital therapeutic as adjunct to treatment with medication in pediatric ADHD. NPJ Digit Med.

[40] Maricich YA, Bickel WK, Marsch LA, Gatchalian K, Botbyl J, Luderer HF (2021). Safety and efficacy of a prescription digital therapeutic as an adjunct to buprenorphine for treatment of opioid use disorder. Curr Med Res Opin.

[41] Emslie G, Kratochvil C, Vitiello B, Silva S, Mayes T, McNulty S, Weller E, Waslick B, Casat C, Walkup J, Pathak S, Rohde P, Posner K, March J, Columbia Suicidality Classification Group, TADS Team (2006). Treatment for Adolescents with Depression Study (TADS): safety results. J Am Acad Child Adolesc Psychiatry.

[42] Treatment for Adolescents With Depression Study Team (2003). Treatment for Adolescents With Depression Study (TADS): rationale, design, and methods. J Am Acad Child Adolesc Psychiatry.

[43] Cheung AH, Zuckerbrot RA, Jensen PS, Laraque D, Stein RE, GLAD-PC Steering Group (2018). Guidelines for adolescent depression in primary care (GLAD-PC): part II. Treatment and ongoing management. Pediatrics.

[44] McCauley E, Gudmundsen G, Schloredt K, Martell C, Rhew I, Hubley S, Dimidjian S (2016). The adolescent behavioral activation program: adapting behavioral activation as a treatment for depression in adolescence. J Clin Child Adolesc Psychol.

[45] Pass L, Lejuez CW, Reynolds S (2018). Brief behavioural activation (brief BA) for adolescent depression: a pilot study. Behav Cogn Psychother.

[46] Lejuez CW, Hopko DR, Acierno R, Daughters SB, Pagoto SL (2011). Ten year revision of the brief behavioral activation treatment for depression: revised treatment manual. Behav Modif.

[47] Kanter JW, Manos RC, Bowe WM, Baruch DE, Busch AM, Rusch LC (2010). What is behavioral activation? A review of the empirical literature. Clin Psychol Rev.

[48] Clarke G, DeBar LL, Pearson JA, Dickerson JF, Lynch FL, Gullion CM, Leo MC (2016). Cognitive behavioral therapy in primary care for youth declining antidepressants: a randomized trial. Pediatrics.

[49] Kambeitz-Ilankovic L, Rzayeva U, Völkel L, Wenzel J, Weiske J, Jessen F, Reininghaus U, Uhlhaas PJ, Alvarez-Jimenez M, Kambeitz J (2022). A systematic review of digital and face-to-face cognitive behavioral therapy for depression. NPJ Digit Med.

[50] Forman-Hoffman VL, Nelson BW, Ranta K, Nazander A, Hilgert O, de Quevedo J (2021). Significant reduction in depressive symptoms among patients with moderately-severe to severe depressive symptoms after participation in a therapist-supported, evidence-based mobile health program delivered via a smartphone app. Internet Interv.

[51] Whittaker R, Stasiak K, McDowell H, Doherty I, Shepherd M, Chua S, Dorey E, Parag V, Ameratunga S, Rodgers A, Merry S (2017). MEMO: an mHealth intervention to prevent the onset of depression in adolescents: a double-blind, randomised, placebo-controlled trial. J Child Psychol Psychiatry.

[52] Fitzpatrick KK, Darcy A, Vierhile M (2017). Delivering cognitive behavior therapy to young adults with symptoms of depression and anxiety using a fully automated conversational agent (Woebot): a randomized controlled trial. JMIR Ment Health.

[53] Kauer SD, Reid SC, Crooke AH, Khor A, Hearps SJ, Jorm AF, Sanci L, Patton G (2012). Self-monitoring using mobile phones in the early stages of adolescent depression: randomized controlled trial. J Med Internet Res.

[54] Kulikov VN, Crosthwaite PC, Hall SA, Flannery JE, Strauss GS, Vierra EM, Koepsell XL, Lake JI, Padmanabhan A (2023). A CBT-based mobile intervention as an adjunct treatment for adolescents with symptoms of depression: a virtual randomized controlled feasibility trial. Front Digit Health.

[55] (2018). Home page. Stanley-Brown: Safety Planning Intervention.

[56] Glen S (2016). Permuted block randomization. Statistics How To.

[57] Kroenke K, Strine TW, Spitzer RL, Williams JB, Berry JT, Mokdad AH (2009). The PHQ-8 as a measure of current depression in the general population. J Affect Disord.

[58] Smith BW, Dalen J, Wiggins K, Tooley E, Christopher P, Bernard J (2008). The brief resilience scale: assessing the ability to bounce back. Int J Behav Med.

[59] Spitzer RL, Kroenke K, Williams JB, Löwe B (2006). A brief measure for assessing generalized anxiety disorder: the GAD-7. Arch Intern Med.

[60] Hays RD, Bjorner JB, Revicki DA, Spritzer KL, Cella D (2009). Development of physical and mental health summary scores from the patient-reported outcomes measurement information system (PROMIS) global items. Qual Life Res.

[61] Eg J, Bilenberg N, Costello EJ, Wesselhoeft R (2018). Self- and parent-reported depressive symptoms rated by the mood and feelings questionnaire. Psychiatry Res.

[62] Brooke J (1996). SUS: a 'quick and dirty' usability scale. Usability Evaluation In Industry.

[63] O’Brien HL, Cairns P, Hall M (2018). A practical approach to measuring user engagement with the refined user engagement scale (UES) and new UES short form. Int J Hum Comput Stud.

[64] ASQ Screening Tool. National Institute of Mental Health (NIMH).

[65] CFR - code of federal regulations. US Department of Food & Drug Administration.

[66] Office of the Commissioner What is a serious adverse event?. US Food and Drug Administration.

[67] Clinical investigation of medical devices for human subjects — good clinical practice. International Organization for Standardization.

[68] R Core Team The R project for statistical computing. The R Foundation.

[69] Little RJ (1988). A test of missing completely at random for multivariate data with missing values. J Am Stat Assoc.

[70] Sterne JA, White IR, Carlin JB, Spratt M, Royston P, Kenward MG, Wood AM, Carpenter JR (2009). Multiple imputation for missing data in epidemiological and clinical research: potential and pitfalls. BMJ.

[71] Schafer JL, Yucel RM (2002). Computational strategies for multivariate linear mixed-effects models with missing values. J Comput Graph Stat.

[72] van Buuren S, Groothuis-Oudshoorn K (2011). mice: multivariate imputation by chained equations in R. J Stat Softw.

[73] Azur MJ, Stuart EA, Frangakis C, Leaf PJ (2011). Multiple imputation by chained equations: what is it and how does it work?. Int J Methods Psychiatr Res.

[74] Bates D, Mächler M, Bolker B, Walker S (2015). Fitting linear mixed-effects models using lme4. J Stat Softw.

[75] Nakagawa S, Schielzeth H (2012). A general and simple method for obtaining R2 from generalized linear mixed-effects models. Methods Ecol Evol.

[76] Nakagawa S, Johnson PC, Schielzeth H (2017). The coefficient of determination R2 and intra-class correlation coefficient from generalized linear mixed-effects models revisited and expanded. J R Soc Interface.

[77] Burnham KP, Anderson DR (2002). Model Selection and Multimodel Inference: A Practical Information-Theoretic Approach.

[78] Kuznetsova A, Brockhoff PB, Christensen RH (2017). lmerTest package: tests in linear mixed effects models. J Stat Soft.

[79] Luke SG (2017). Evaluating significance in linear mixed-effects models in R. Behav Res Methods.

[80] Jaeschke R, Singer J, Guyatt GH (1989). Measurement of health status. Ascertaining the minimal clinically important difference. Control Clin Trials.

[81] Coley RY, Boggs JM, Beck A, Hartzler AL, Simon GE (2020). Defining success in measurement-based care for depression: a comparison of common metrics. Psychiatr Serv.

[82] Baumel A, Muench F, Edan S, Kane JM (2019). Objective user engagement with mental health apps: systematic search and panel-based usage analysis. J Med Internet Res.

[83] Wu A, Scult MA, Barnes ED, Betancourt JA, Falk A, Gunning FM (2021). Smartphone apps for depression and anxiety: a systematic review and meta-analysis of techniques to increase engagement. NPJ Digit Med.

[84] Richards D, Enrique A, Eilert N, Franklin M, Palacios J, Duffy D, Earley C, Chapman J, Jell G, Sollesse S, Timulak L (2020). A pragmatic randomized waitlist-controlled effectiveness and cost-effectiveness trial of digital interventions for depression and anxiety. NPJ Digit Med.

[85] Ritterband LM, Thorndike FP, Ingersoll KS, Lord HR, Gonder-Frederick L, Frederick C, Quigg MS, Cohn WF, Morin CM (2017). Effect of a web-based cognitive behavior therapy for insomnia intervention with 1-year follow-up: a randomized clinical trial. JAMA Psychiatry.

[86] Clarke AM, Kuosmanen T, Barry MM (2015). A systematic review of online youth mental health promotion and prevention interventions. J Youth Adolesc.

[87] Sauro J (2011). Practical guide to the system usability scale background, benchmarks, and best practices. Scientific Research Publishing.

[88] Noyes BK, Munoz DP, Khalid-Khan S, Brietzke E, Booij L (2022). Is subthreshold depression in adolescence clinically relevant?. J Affect Disord.

[89] van Straten A, Seekles W, van 't Veer-Tazelaar NJ, Beekman AT, Cuijpers P (2010). Stepped care for depression in primary care: what should be offered and how?. Med J Aust.

[90] Thase ME, Wright JH, Eells TD, Barrett MS, Wisniewski SR, Balasubramani GK, McCrone P, Brown GK (2018). Improving the efficiency of psychotherapy for depression: computer-assisted versus standard CBT. Am J Psychiatry.

[91] Williams ME, Latta J, Conversano P (2008). Eliminating the wait for mental health services. J Behav Health Serv Res.

[92] Kowalewski K, McLennan JD, McGrath PJ (2011). A preliminary investigation of wait times for child and adolescent mental health services in Canada. J Can Acad Child Adolesc Psychiatry.

