# Supplementary material for: Preliminary Efficacy of a Digital Intervention for Adolescent Depression: Randomized Controlled Trial
Source: J Med Internet Res. 2024 Feb 7;26:e48467. doi: 10.2196/48467 (PMC10882470; doi:10.2196/48467)
Supplement: Multimedia Appendix 1 [file jmir_v26i1e48467_app1.docx]

**Table 1.** Missing data analysis by cohort.

|  | Moderate-to-severe cohort | Mild-to-severe cohort |
| --- | --- | --- |
| Effect of Spark version | χ^2^=0.183; *P*=.70  Missing versus no missing  Spark 2.1: 19 versus 43  Spark 2.2: 16 versus 53 | χ^2^=1.041; , *P*=.38  Missing vs.versus no missing  Spark 2.1: 25 versus 52  Spark 2.2: 19 versus 57 |
| Effect of group | χ^2^=5.375; *P*=.03^*^  Missing versus no missing  Treatment: 24 versus 39  Control: 11 versus 47 | χ^2^=5.767; *P*=.02^*^  Missing versus no missing  Treatment: 28 versus 46  Control: 16 versus 63 |
| Effect of week | χ^2^=43.099; *P* <.001^***^  Missing versus no missing:  W0: 0 versus 121  W1: 7 versus 114  W2: 12 versus 109  W 3: 12 versus 109  W4: 20 versus 101  W5: 29 versus 92 | χ^2^=50.962; *P* <.001^***^  Missing versus no missing:  W0: 0 versus 153  W1: 10 versus 143  W2: 13 versus 140  W 3: 16 versus 137  W4: 22 versus 131  W5: 36 versus 117 |
| Group×week | χ^2^=2.309; *P*=.68  W0 W1 W2 W3 W4 W5  T: 0 4 6 9 14 20  C: 0 3 6 3 6 9 | χ^2^=2.314; *P*=.70  W0 W1 W2 W3 W4 W5  T: 0 5 7 11 16 23  C: 0 5 6 5 6 13 |
| Baseline severity | χ^2^=.091; *P*=.97  Missing versus no missing  Moderate: 15 versus 37  Moderate-to-severe: 15 versus 35  Severe: 5 vs.versus 14 | χ^2^=.091; *P*=.97  Missing versus no missing  Mild: 9 versus 23  Moderate: 15 versus 37  Moderate-to-severe: 15 versus 35  Severe: 5 versus 14 |
| Baseline severity×week | χ^2^=4.482; *P*=.82  W0 W1 W2 W 3 W4 W5  Mod: 0 5 5 7 9 11  Mo-S: 0 1 5 3 9 13  Sev: 0 1 2 2 2 5 | χ^2^=4.482; *P*=.85  W0 W1 W2 W 3 W4 W5  Mild: 0 3 1 4 2 7  Mod: 0 5 5 7 9 11  Mi-S: 0 1 5 3 9 13  Sev: 0 1 2 2 2 5 |
| Effect of age group | χ^2^=6.545; *P*=.02^*^  Missing versus no missing  Age 13 to 17 years: 26 versus 52  Age ≥18 years+: 9 versus 44 | χ^2^=6.846; *P*=.02^*^  Missing versus no missing  Age 13 to 17 years: 32 versus 54  Age ≥18 years+: 12 versus 55 |
| Effect of gender | χ^2^=3.757; *P*=.17  Missing versus no missing  Female: 19 versus 62  Male: 11 versus 15  Non-binary: 9 versus 5 | χ^2^=2.712; *P*=.26  Missing versus no missing  Female: 24 versus 74  Male: 15 versus 24  Non-binary: 5 versus 11 |

Note: W0=Baseline, W1=Week 1, W2=Week 2, W3=Week 3, W4=Week 4, W5=Week 5, T = Treatment, C = Control , Mod = Moderate, Mo-S = moderate-to-severe, Mi-S = mild-to-severe, Sev = Severe
