# Supplementary material for: Preliminary Efficacy of a Digital Intervention for Adolescent Depression: Randomized Controlled Trial
Source: J Med Internet Res. 2024 Feb 7;26:e48467. doi: 10.2196/48467 (PMC10882470; doi:10.2196/48467)
Supplement: Multimedia Appendix 2 [file jmir_v26i1e48467_app2.pdf]

CONSORT-EHEALTH (V 1.6.1) - Submission/Publication Form

The CONSORT-EHEALTH checklist is intended for authors of randomized trials evaluating web-based and Internet-based applications/interventions, including mobile interventions, electronic games (incl multiplayer games), social media, certain telehealth applications, and other interactive and/or networked electronic applications. Some of the items (e.g. all subitems under item 5 - description of the intervention) may also be applicable for other study designs.

The goal of the CONSORT EHEALTH checklist and guideline is to be
a) a guide for reporting for authors of RCTs,
b) to form a basis for appraisal of an ehealth trial (in terms of validity)

CONSORT-EHEALTH items/subitems are MANDATORY reporting items for studies published in the Journal of Medical Internet Research and other journals / scientific societies endorsing the checklist.

Items numbered 1., 2., 3., 4a., 4b etc are original CONSORT or CONSORT-NPT (non-pharmacologic treatment) items.
Items with Roman numerals (i., ii, iii, iv etc.) are CONSORT-EHEALTH extensions/clarifications.

As the CONSORT-EHEALTH checklist is still considered in a formative stage, we would ask that you also RATE ON A SCALE OF 1-5 how important/useful you feel each item is FOR THE PURPOSE OF THE CHECKLIST and reporting guideline (optional).

Mandatory reporting items are marked with a red *.
In the textboxes, either copy & paste the relevant sections from your manuscript into this form - please include any quotes from your manuscript in QUOTATION MARKS,
or answer directly by providing additional information not in the manuscript, or elaborating on why the item was not relevant for this study.

YOUR ANSWERS WILL BE PUBLISHED AS A SUPPLEMENTARY FILE TO YOUR PUBLICATION IN JMIR AND ARE CONSIDERED PART OF YOUR PUBLICATION (IF ACCEPTED).
Please fill in these questions diligently. Information will not be copyedited, so please use proper spelling and grammar, use correct capitalization, and avoid abbreviations.

DO NOT FORGET TO SAVE AS PDF _AND_ CLICK THE SUBMIT BUTTON SO YOUR ANSWERS ARE IN OUR DATABASE !!!

Citation Suggestion (if you append the pdf as Appendix we suggest to cite this paper in the caption):
Eysenbach G, CONSORT-EHEALTH Group

CONSORT-EHEALTH: Improving and Standardizing Evaluation Reports of Web-based and Mobile Health Interventions
J Med Internet Res 2011;13(4):e126
URL: http://www.jmir.org/2011/4/e126/
doi: 10.2196/jmir.1923
PMID: 22209829

emily.peake@bighealth.com Switch account

Not shared

Draft saved

* Indicates required question

Your name *

First Last

Emily Peake

Primary Affiliation (short), City, Country *

University of Toronto, Toronto, Canada

Big Health, Inc. San Francisco, USA

Your e-mail address *

abc@gmail.com

emily.peake@bighealth.com

Title of your manuscript *

Provide the (draft) title of your manuscript.

Preliminary Efficacy of a Digital Intervention for Adolescent Depression: Randomized Controlled Trial

Name of your App/Software/Intervention *

If there is a short and a long/alternate name, write the short name first and add the long name in brackets.

Spark

Evaluated Version (if any)

e.g. "V1", "Release 2017-03-01", "Version 2.0.27913"

Spark V 2.1 & 2.2

Language(s) *

What language is the intervention/app in? If multiple languages are available, separate by comma (e.g. "English, French")

English

URL of your Intervention Website or App

e.g. a direct link to the mobile app on app in appstore (itunes, Google Play), or URL of the website. If the intervention is a DVD or hardware, you can also link to an Amazon page.

Your answer

URL of an image/screenshot (optional)

Your answer

Accessibility *

Can an enduser access the intervention presently?

- ○ access is free and open
- ○ access only for special usergroups, not open
- ○ access is open to everyone, but requires payment/subscription/in-app purchases
- ◉ app/intervention no longer accessible
- ○ Other:

Primary Medical Indication/Disease/Condition *

e.g. "Stress", "Diabetes", or define the target group in brackets after the condition, e.g. "Autism (Parents of children with)", "Alzheimers (Informal Caregivers of)"

Depression

Primary Outcomes measured in trial *

comma-separated list of primary outcomes reported in the trial

PHQ-8

Secondary/other outcomes

Are there any other outcomes the intervention is expected to affect?

GAD-7, PROMIS Pediatric Global 7+2, MFQ-PS

Recommended "Dose" *

What do the instructions for users say on how often the app should be used?

- ○ Approximately Daily
- ◉ Approximately Weekly
- ○ Approximately Monthly
- ○ Approximately Yearly
- ○ "as needed"
- ○ Other:

Approx. Percentage of Users (starters) still using the app as recommended after 3 months *

- ◉ unknown / not evaluated
- ○ 0-10%
- ○ 11-20%
- ○ 21-30%
- ○ 31-40%
- ○ 41-50%
- ○ 51-60%
- ○ 61-70%
- ○ 71%-80%
- ○ 81-90%
- ○ 91-100%
- ○ Other:

Overall, was the app/intervention effective? *

- ( ) yes: all primary outcomes were significantly better in intervention group vs control
- (•) partly: SOME primary outcomes were significantly better in intervention group vs control
- ( ) no statistically significant difference between control and intervention
- ( ) potentially harmful: control was significantly better than intervention in one or more outcomes
- ( ) inconclusive: more research is needed
- ( ) Other:

Article Preparation Status/Stage *

At which stage in your article preparation are you currently (at the time you fill in this form)

- ( ) not submitted yet - in early draft status
- ( ) not submitted yet - in late draft status, just before submission
- ( ) submitted to a journal but not reviewed yet
- ( ) submitted to a journal and after receiving initial reviewer comments
- (•) submitted to a journal and accepted, but not published yet
- ( ) published
- ( ) Other:

Journal *

If you already know where you will submit this paper (or if it is already submitted), please provide the journal name (if it is not JMIR, provide the journal name under "other")

- ○ not submitted yet / unclear where I will submit this
- ◉ Journal of Medical Internet Research (JMIR)
- ○ JMIR mHealth and UHealth
- ○ JMIR Serious Games
- ○ JMIR Mental Health
- ○ JMIR Public Health
- ○ JMIR Formative Research
- ○ Other JMIR sister journal
- ○ Other:

Is this a full powered effectiveness trial or a pilot/feasibility trial? *

- ◉ Pilot/feasibility
- ○ Fully powered

Manuscript tracking number *

If this is a JMIR submission, please provide the manuscript tracking number under "other" (The ms tracking number can be found in the submission acknowledgement email, or when you login as author in JMIR. If the paper is already published in JMIR, then the ms tracking number is the four-digit number at the end of the DOI, to be found at the bottom of each published article in JMIR)

- ○ no ms number (yet) / not (yet) submitted to / published in JMIR
- ◉ Other: JMIR ms#48467

TITLE AND ABSTRACT

1a) TITLE: Identification as a randomized trial in the title

1a) Does your paper address CONSORT item 1a? *

I.e does the title contain the phrase "Randomized Controlled Trial"? (if not, explain the reason under "other")

- (x) yes
- ( ) Other:

1a-i) Identify the mode of delivery in the title

Identify the mode of delivery. Preferably use “web-based” and/or “mobile” and/or “electronic game” in the title. Avoid ambiguous terms like “online”, “virtual”, “interactive”. Use “Internet-based” only if Intervention includes non-web-based Internet components (e.g. email), use “computer-based” or “electronic” only if offline products are used. Use “virtual” only in the context of “virtual reality” (3-D worlds). Use “online” only in the context of “online support groups”. Complement or substitute product names with broader terms for the class of products (such as “mobile” or “smart phone” instead of “iphone”), especially if the application runs on different platforms.

	1	2	3	4	5	
subitem not at all important	( )	( )	( )	( )	( )	essential

Does your paper address subitem 1a-i? *

Copy and paste relevant sections from manuscript title (include quotes in quotation marks "like this" to indicate direct quotes from your manuscript), or elaborate on this item by providing additional information not in the ms, or briefly explain why the item is not applicable/relevant for your study

"Preliminary Efficacy of a Digital Intervention"

1a-ii) Non-web-based components or important co-interventions in title

Mention non-web-based components or important co-interventions in title, if any (e.g., “with telephone support”).

	1	2	3	4	5	
subitem not at all important	○	○	○	○	○	essential

Does your paper address subitem 1a-ii?

Copy and paste relevant sections from manuscript title (include quotes in quotation marks "like this" to indicate direct quotes from your manuscript), or elaborate on this item by providing additional information not in the ms, or briefly explain why the item is not applicable/relevant for your study

Your answer

1a-iii) Primary condition or target group in the title

Mention primary condition or target group in the title, if any (e.g., “for children with Type I Diabetes”) Example: A Web-based and Mobile Intervention with Telephone Support for Children with Type I Diabetes: Randomized Controlled Trial

	1	2	3	4	5	
subitem not at all important	○	○	○	○	○	essential

Does your paper address subitem 1a-iii? *

Copy and paste relevant sections from manuscript title (include quotes in quotation marks "like this" to indicate direct quotes from your manuscript), or elaborate on this item by providing additional information not in the ms, or briefly explain why the item is not applicable/relevant for your study

"Digital Intervention for Adolescent Depression"

1b) ABSTRACT: Structured summary of trial design, methods, results, and conclusions

NPT extension: Description of experimental treatment, comparator, care providers, centers, and blinding status.

1b-i) Key features/functionalities/components of the intervention and comparator in the METHODS section of the ABSTRACT

Mention key features/functionalities/components of the intervention and comparator in the abstract. If possible, also mention theories and principles used for designing the site. Keep in mind the needs of systematic reviewers and indexers by including important synonyms. (Note: Only report in the abstract what the main paper is reporting. If this information is missing from the main body of text, consider adding it)

	1	2	3	4	5	
subitem not at all important	○	○	○	○	○	essential

Does your paper address subitem 1b-i? *

Copy and paste relevant sections from the manuscript abstract (include quotes in quotation marks "like this" to indicate direct quotes from your manuscript), or elaborate on this item by providing additional information not in the ms, or briefly explain why the item is not applicable/relevant for your study

“To examine the efficacy of a mobile app DTx vs. an active control as an adjunct treatment for adolescent depression symptoms.” “A virtual, open-label, randomized clinical trial was conducted nationwide with a partial cross-over design”. “Participants were randomized (1:1) to the cognitive behavioral therapy (CBT)-based treatment app (Spark), or to a psychoeducational control app (Control) to use for 5 weeks.”

1b-ii) Level of human involvement in the METHODS section of the ABSTRACT

Clarify the level of human involvement in the abstract, e.g., use phrases like “fully automated” vs. “therapist/nurse/care provider/physician-assisted” (mention number and expertise of providers involved, if any). (Note: Only report in the abstract what the main paper is reporting. If this information is missing from the main body of text, consider adding it)

	1	2	3	4	5	
subitem not at all important	○	○	○	○	○	essential

Does your paper address subitem 1b-ii?

Copy and paste relevant sections from the manuscript abstract (include quotes in quotation marks "like this" to indicate direct quotes from your manuscript), or elaborate on this item by providing additional information not in the ms, or briefly explain why the item is not applicable/relevant for your study

“mobile app DTx” “self-guided, CBT-based DTx intervention”

1b-iii) Open vs. closed, web-based (self-assessment) vs. face-to-face assessments in the METHODS section of the ABSTRACT

Mention how participants were recruited (online vs. offline), e.g., from an open access website or from a clinic or a closed online user group (closed usergroup trial), and clarify if this was a purely web-based trial, or there were face-to-face components (as part of the intervention or for assessment). Clearly say if outcomes were self-assessed through questionnaires (as common in web-based trials). Note: In traditional offline trials, an open trial (open-label trial) is a type of clinical trial in which both the researchers and participants know which treatment is being administered. To avoid confusion, use “blinded” or “unblinded” to indicated the level of blinding instead of “open”, as “open” in web-based trials usually refers to “open access” (i.e. participants can self-enrol). (Note: Only report in the abstract what the main paper is reporting. If this information is missing from the main body of text, consider adding it)

	1	2	3	4	5	
subitem not at all important	○	○	○	○	○	essential

Does your paper address subitem 1b-iii?

Copy and paste relevant sections from the manuscript abstract (include quotes in quotation marks "like this" to indicate direct quotes from your manuscript), or elaborate on this item by providing additional information not in the ms, or briefly explain why the item is not applicable/relevant for your study

"An internet-based open-label randomized control trial was conducted nationwide with a partial crossover design, and 168 adolescents aged 13 to 21 years with symptoms of depression were recruited between November 2020 and September 2021." and "The primary outcome was a between-group (Spark vs control) difference in the change in self-reported depression symptoms from baseline to postintervention, as measured by the Patient Health Questionnaire-8 (PHQ-8) using a linear mixed-effects analysis"

1b-iv) RESULTS section in abstract must contain use data

Report number of participants enrolled/assessed in each group, the use/uptake of the intervention (e.g., attrition/adherence metrics, use over time, number of logins etc.), in addition to primary/secondary outcomes. (Note: Only report in the abstract what the main paper is reporting. If this information is missing from the main body of text, consider adding it)

	1	2	3	4	5	
subitem not at all important	○	○	○	○	○	essential

Does your paper address subitem 1b-iv?

Copy and paste relevant sections from the manuscript abstract (include quotes in quotation marks "like this" to indicate direct quotes from your manuscript), or elaborate on this item by providing additional information not in the ms, or briefly explain why the item is not applicable/relevant for your study

Your answer

1b-v) CONCLUSIONS/DISCUSSION in abstract for negative trials

Conclusions/Discussions in abstract for negative trials: Discuss the primary outcome - if the trial is negative (primary outcome not changed), and the intervention was not used, discuss whether negative results are attributable to lack of uptake and discuss reasons. (Note: Only report in the abstract what the main paper is reporting. If this information is missing from the main body of text, consider adding it)

	1	2	3	4	5	
subitem not at all important	○	○	○	○	○	essential

Does your paper address subitem 1b-v?

Copy and paste relevant sections from the manuscript abstract (include quotes in quotation marks "like this" to indicate direct quotes from your manuscript), or elaborate on this item by providing additional information not in the ms, or briefly explain why the item is not applicable/relevant for your study

"There is initial evidence that a self-guided, cognitive behavioral therapy–based DTx intervention may effectively treat mild to severe depression symptoms in adolescents."

INTRODUCTION

2a) In INTRODUCTION: Scientific background and explanation of rationale

2a-i) Problem and the type of system/solution

Describe the problem and the type of system/solution that is object of the study: intended as stand-alone intervention vs. incorporated in broader health care program? Intended for a particular patient population? Goals of the intervention, e.g., being more cost-effective to other interventions, replace or complement other solutions? (Note: Details about the intervention are provided in “Methods” under 5)

	1	2	3	4	5	
subitem not at all important	○	○	○	○	○	essential

Does your paper address subitem 2a-i? *

Copy and paste relevant sections from the manuscript (include quotes in quotation marks "like this" to indicate direct quotes from your manuscript), or elaborate on this item by providing additional information not in the ms, or briefly explain why the item is not applicable/relevant for your study

“Depression is a pervasive mental health disorder affecting nearly four million adolescents in the USA, with this number expected to grow annually [1–4]. Depression can be lifelong, often emerging in adolescence and increasing the risk for poor physical health, social and relational problems, academic and employment difficulties, and reduced overall well-being [5–7]. Despite a growing need for effective treatment options, adolescents access [3,6] and adhere to [8,9] mental health treatment at alarmingly low rates due to a number of factors. Stigma and a general difficulty identifying symptoms of a mental illness inhibit adolescents’ willingness or awareness to seek treatment [10,11]. When they do seek help, adolescents face months-long waitlists [16].Other external forces drive the unmet clinical needs of adolescents experiencing depression, including costs [2,12], a widening divide in the accessibility of treatment [13,14], and an outstripped supply of mental health professionals [13,15]. Barriers in access to mental health treatment and depression incidence rates in adolescents have been exacerbated by the COVID-19 pandemic, necessitating action and new solutions [17–21].

Digital health programs, delivered via smartphones and computers, may serve as an accessible way to provide treatment options for mental health conditions, including depression [22–29], although limitations exist including a lack of evidence-based protocols and clinical validation [24,25,27]. Digital therapeutics (DTx), which are evidence-based software programs designed to prevent, treat, or manage health conditions, serve to address these limitations[30]. Smartphone-based DTx are particularly well-suited for adolescents given their technological literacy, ubiquitous cell phone use, and privacy preferences [22,29,31,32]. DTx are also easily scalable, can serve as adjunct to standard care, improve treatment fidelity [33–35], and be cost-effective [36] These technologies have been shown to be preliminarily effective in treating adolescents with a variety of mental health concerns including attention-deficit/hyperactivity disorder (ADHD)[37] , sleep disorders, eating disorders and anxiety[38]. Ultimately, DTx fill critical gaps of non-DTx digital treatments and can help patients receive effective, data-driven treatment with the added benefit of home access at any time [35,39,40].

Cognitive behavioral therapy (CBT) is a widely recognized and recommended treatment for depression and considered the standard of care for adolescents [41–43]. Behavioral activation (BA), an important component of CBT [44–47], involves engaging in value-based behaviors that are rewarding or elicit a sense of mastery to help reduce depression symptoms using a combination of motivational strategies, reward-seeking, and natural reinforcers, alongside reducing maladaptive, avoidant behavior. BA has been shown to be an effective stand-alone treatment for adolescent depression [44–48]. Digital CBT applications have demonstrated promise in treating depression [23,26,49,50], and a BA-focused DTx may represent an effective and accessible treatment option for adolescents.

Limitations such as low user engagement and insufficient existing clinical validation (e.g. randomized controlled trials) may limit widespread adoption of DTx [24,25,27,49]. To our knowledge, there are no DTx for depression tailored specifically for adolescents, including BA-based DTx, and limited evidence exists regarding the efficacy of a smartphone-based DTx for adolescent depression [51–53].

The present study evaluates the efficacy of Spark, a 5-week self-guided mobile app intervention based on validated BA treatment protocols for adolescents with depression [44,45]. Adolescents aged 13-21 were recruited nationwide into a virtual randomized clinical

[44,45]. Adolescents aged 13-21 were recruited nationwide into a virtual randomized clinical trial (RCT) comparing Spark to an active control. Our main goal was to assess the preliminary efficacy of Spark in treating moderate to severe depression symptoms as an adjunct to usual care, by measuring group differences in 1) changes in depression symptoms over time, including whether each group had a minimally clinically important decrease in depression symptoms; 2) the proportion of participants who achieved remission; and 3) the proportion of participants who demonstrated treatment response. As this RCT was conducted between November 2020 and September 2021, another study goal was to provide mental health resources to adolescents experiencing symptoms of depression during the COVID-19 pandemic."

2a-ii) Scientific background, rationale: What is known about the (type of) system

Scientific background, rationale: What is known about the (type of) system that is the object of the study (be sure to discuss the use of similar systems for other conditions/diagnoses, if appropiate), motivation for the study, i.e. what are the reasons for and what is the context for this specific study, from which stakeholder viewpoint is the study performed, potential impact of findings [2]. Briefly justify the choice of the comparator.

	1	2	3	4	5	
subitem not at all important	○	○	○	○	○	essential

Does your paper address subitem 2a-ii? *

Copy and paste relevant sections from the manuscript (include quotes in quotation marks "like this" to indicate direct quotes from your manuscript), or elaborate on this item by providing additional information not in the ms, or briefly explain why the item is not applicable/relevant for your study

“The present study evaluates the efficacy of Spark, a 5-week self-guided mobile app intervention based on validated BA treatment protocols for adolescents with depression [44,45]. Adolescents aged 13-21 were recruited nationwide into a virtual randomized clinical trial (RCT) comparing Spark to an active control. Our main goal was to assess the preliminary efficacy of Spark in treating moderate to severe depression symptoms as an adjunct to usual care, by measuring group differences in 1) changes in depression symptoms over time, including whether each group had a minimally clinically important decrease in depression symptoms; 2) the proportion of participants who achieved remission; and 3) the proportion of participants who demonstrated treatment response. As this RCT was conducted between November 2020 and September 2021, another study goal was to provide mental health resources to adolescents experiencing symptoms of depression during the COVID-19 pandemic.”
The active control app “contained 5 modules of age-appropriate psychoeducational content related to the neurobiology of depression and did not contain any active CBT or BA components.” As psychoeducation is often used as a form of treatment for depression, this served as a strong comparator to assess the effects of the core active ingredients in Spark.

2b) In INTRODUCTION: Specific objectives or hypotheses

Does your paper address CONSORT subitem 2b? *

Copy and paste relevant sections from the manuscript (include quotes in quotation marks "like this" to indicate direct quotes from your manuscript), or elaborate on this item by providing additional information not in the ms, or briefly explain why the item is not applicable/relevant for your study

“Our main goal was to assess the preliminary efficacy of Spark in treating moderate to severe depression symptoms as an adjunct to usual care, by measuring group differences in 1) changes in depression symptoms over time, including whether each group had a minimally clinically important decrease in depression symptoms; 2) the proportion of participants who achieved remission; and 3) the proportion of participants who demonstrated treatment response. As this RCT was conducted between November 2020 and September 2021, another study goal was to provide mental health resources to adolescents experiencing symptoms of depression during the COVID-19 pandemic.”

METHODS

3a) Description of trial design (such as parallel, factorial) including allocation ratio

Does your paper address CONSORT subitem 3a? *

Copy and paste relevant sections from the manuscript (include quotes in quotation marks "like this" to indicate direct quotes from your manuscript), or elaborate on this item by providing additional information not in the ms, or briefly explain why the item is not applicable/relevant for your study

“This open-label, partial-crossover RCT compared Spark to an app with psychoeducational content (control app) in adolescents aged 13-21 with self-identified depression symptoms.” “Participants were randomized 1:1 to the Spark or Control arm using a block randomization approach ranging in multiples of two from 6-12 [56].”

3b) Important changes to methods after trial commencement (such as eligibility criteria), with reasons

Does your paper address CONSORT subitem 3b? *

Copy and paste relevant sections from the manuscript (include quotes in quotation marks "like this" to indicate direct quotes from your manuscript), or elaborate on this item by providing additional information not in the ms, or briefly explain why the item is not applicable/relevant for your study

No major changes were made to methods after trial commencement.

3b-i) Bug fixes, Downtimes, Content Changes

Bug fixes, Downtimes, Content Changes: ehealth systems are often dynamic systems. A description of changes to methods therefore also includes important changes made on the intervention or comparator during the trial (e.g., major bug fixes or changes in the functionality or content) (5-iii) and other “unexpected events” that may have influenced study design such as staff changes, system failures/downtimes, etc. [2].

	1	2	3	4	5	
subitem not at all important	○	○	○	○	○	essential

Does your paper address subitem 3b-i?

Copy and paste relevant sections from the manuscript (include quotes in quotation marks "like this" to indicate direct quotes from your manuscript), or elaborate on this item by providing additional information not in the ms, or briefly explain why the item is not applicable/relevant for your study

Your answer

4a) Eligibility criteria for participants

Does your paper address CONSORT subitem 4a? *

Copy and paste relevant sections from the manuscript (include quotes in quotation marks "like this" to indicate direct quotes from your manuscript), or elaborate on this item by providing additional information not in the ms, or briefly explain why the item is not applicable/relevant for your study

“ The following inclusion criteria were applied: (1) aged 13 to 21; (2) self-reported symptoms of depression; (3) residing in the USA for the duration of the 5-week study; (4) under the care of a US-based primary care and/or licensed mental healthcare provider and willing and able to provide the name and contact information of the provider during consent appointment; (5) English fluency and literacy of adolescent and consenting legal guardian if under 18; (6) access to a smartphone (iPhone 5s or later or running Android 4.4 KitKat or later) and regular internet access; (7) willing to provide informed e-consent/assent and have legal guardian willing to provide informed e-consent if under 18. After phase one and before phase two, one additional inclusion criteria was added: (8) stable for at least two months on any treatment (including medication or psychotherapy) for a mental health disorder. Participants were excluded if they self-reported any of the following: (1) self-reported lifetime suicide attempt or active self-harm or active suicidal ideation with intent; (2) have a diagnosis by a clinician of bipolar disorder, substance use disorder, or any psychotic disorder including schizophrenia; (3) incapable of understanding or completing study procedures and digital intervention as determined by participant, patient/legal guardian, healthcare provider, or clinical research team. After phase one and before phase two, one additional exclusion criteria was added: (4) previously participated in user testing or clinical testing of the Spark app.“

4a-i) Computer / Internet literacy

Computer / Internet literacy is often an implicit “de facto” eligibility criterion - this should be explicitly clarified.

	1	2	3	4	5	
subitem not at all important	○	○	○	○	○	essential

Does your paper address subitem 4a-i?

Copy and paste relevant sections from the manuscript (include quotes in quotation marks "like this" to indicate direct quotes from your manuscript), or elaborate on this item by providing additional information not in the ms, or briefly explain why the item is not applicable/relevant for your study

Your answer

4a-ii) Open vs. closed, web-based vs. face-to-face assessments:

Open vs. closed, web-based vs. face-to-face assessments: Mention how participants were recruited (online vs. offline), e.g., from an open access website or from a clinic, and clarify if this was a purely web-based trial, or there were face-to-face components (as part of the intervention or for assessment), i.e., to what degree got the study team to know the participant. In online-only trials, clarify if participants were quasi-anonymous and whether having multiple identities was possible or whether technical or logistical measures (e.g., cookies, email confirmation, phone calls) were used to detect/prevent these.

	1	2	3	4	5	
subitem not at all important	○	○	○	○	○	essential

Does your paper address subitem 4a-ii? *

Copy and paste relevant sections from the manuscript (include quotes in quotation marks "like this" to indicate direct quotes from your manuscript), or elaborate on this item by providing additional information not in the ms, or briefly explain why the item is not applicable/relevant for your study

“Participants were recruited nationwide via online advertising and word of mouth.”

4a-iii) Information giving during recruitment

Information given during recruitment. Specify how participants were briefed for recruitment and in the informed consent procedures (e.g., publish the informed consent documentation as appendix, see also item X26), as this information may have an effect on user self-selection, user expectation and may also bias results.

	1	2	3	4	5	
subitem not at all important	○	○	○	○	○	essential

Does your paper address subitem 4a-iii?

Copy and paste relevant sections from the manuscript (include quotes in quotation marks "like this" to indicate direct quotes from your manuscript), or elaborate on this item by providing additional information not in the ms, or briefly explain why the item is not applicable/relevant for your study

Your answer

4b) Settings and locations where the data were collected

Does your paper address CONSORT subitem 4b? *

Copy and paste relevant sections from the manuscript (include quotes in quotation marks "like this" to indicate direct quotes from your manuscript), or elaborate on this item by providing additional information not in the ms, or briefly explain why the item is not applicable/relevant for your study

The study was virtual. Participants and study staff could be located anywhere in the continental United States, Hawaii, and Alaska. The study sponsor, Limbix Health, Inc., was headquartered in San Francisco, California.

4b-i) Report if outcomes were (self-)assessed through online questionnaires

Clearly report if outcomes were (self-)assessed through online questionnaires (as common in web-based trials) or otherwise.

	1	2	3	4	5	
subitem not at all important	○	○	○	○	○	essential

Does your paper address subitem 4b-i? *

Copy and paste relevant sections from the manuscript (include quotes in quotation marks "like this" to indicate direct quotes from your manuscript), or elaborate on this item by providing additional information not in the ms, or briefly explain why the item is not applicable/relevant for your study

All outcomes were assessed through online questionnaires either via the mobile app used for the study or through a web portal.

4b-ii) Report how institutional affiliations are displayed

Report how institutional affiliations are displayed to potential participants [on ehealth media], as affiliations with prestigious hospitals or universities may affect volunteer rates, use, and reactions with regards to an intervention.(Not a required item – describe only if this may bias results)

	1	2	3	4	5	
subitem not at all important	○	○	○	○	○	essential

Does your paper address subitem 4b-ii?

Copy and paste relevant sections from the manuscript (include quotes in quotation marks "like this" to indicate direct quotes from your manuscript), or elaborate on this item by providing additional information not in the ms, or briefly explain why the item is not applicable/relevant for your study

Your answer

5) The interventions for each group with sufficient details to allow replication, including how and when they were actually administered

5-i) Mention names, credential, affiliations of the developers, sponsors, and owners

Mention names, credential, affiliations of the developers, sponsors, and owners [6] (if authors/evaluators are owners or developer of the software, this needs to be declared in a “Conflict of interest” section or mentioned elsewhere in the manuscript).

	1	2	3	4	5	
subitem not at all important	○	○	○	○	○	essential

Does your paper address subitem 5-i?

Copy and paste relevant sections from the manuscript (include quotes in quotation marks "like this" to indicate direct quotes from your manuscript), or elaborate on this item by providing additional information not in the ms, or briefly explain why the item is not applicable/relevant for your study

Your answer

5-ii) Describe the history/development process

Describe the history/development process of the application and previous formative evaluations (e.g., focus groups, usability testing), as these will have an impact on adoption/use rates and help with interpreting results.

	1	2	3	4	5	
subitem not at all important	○	○	○	○	○	essential

Does your paper address subitem 5-ii?

Copy and paste relevant sections from the manuscript (include quotes in quotation marks "like this" to indicate direct quotes from your manuscript), or elaborate on this item by providing additional information not in the ms, or briefly explain why the item is not applicable/relevant for your study

Your answer

5-iii) Revisions and updating

Revisions and updating. Clearly mention the date and/or version number of the application/intervention (and comparator, if applicable) evaluated, or describe whether the intervention underwent major changes during the evaluation process, or whether the development and/or content was “frozen” during the trial. Describe dynamic components such as news feeds or changing content which may have an impact on the replicability of the intervention (for unexpected events see item 3b).

	1	2	3	4	5	
subitem not at all important	○	○	○	○	○	essential

Does your paper address subitem 5-iii?

Copy and paste relevant sections from the manuscript (include quotes in quotation marks "like this" to indicate direct quotes from your manuscript), or elaborate on this item by providing additional information not in the ms, or briefly explain why the item is not applicable/relevant for your study

Your answer

5-iv) Quality assurance methods

Provide information on quality assurance methods to ensure accuracy and quality of information provided [1], if applicable.

	1	2	3	4	5	
subitem not at all important	○	○	○	○	○	essential

Does your paper address subitem 5-iv?

Copy and paste relevant sections from the manuscript (include quotes in quotation marks "like this" to indicate direct quotes from your manuscript), or elaborate on this item by providing additional information not in the ms, or briefly explain why the item is not applicable/relevant for your study

Your answer

5-v) Ensure replicability by publishing the source code, and/or providing screenshots/screen-capture video, and/or providing flowcharts of the algorithms used

Ensure replicability by publishing the source code, and/or providing screenshots/screen-capture video, and/or providing flowcharts of the algorithms used. Replicability (i.e., other researchers should in principle be able to replicate the study) is a hallmark of scientific reporting.

	1	2	3	4	5	
subitem not at all important	○	○	○	○	○	essential

Does your paper address subitem 5-v?

Copy and paste relevant sections from the manuscript (include quotes in quotation marks "like this" to indicate direct quotes from your manuscript), or elaborate on this item by providing additional information not in the ms, or briefly explain why the item is not applicable/relevant for your study

Your answer

5-vi) Digital preservation

Digital preservation: Provide the URL of the application, but as the intervention is likely to change or disappear over the course of the years; also make sure the intervention is archived (Internet Archive, webcitation.org, and/or publishing the source code or screenshots/videos alongside the article). As pages behind login screens cannot be archived, consider creating demo pages which are accessible without login.

	1	2	3	4	5	
subitem not at all important	○	○	○	○	○	essential

Does your paper address subitem 5-vi?

Copy and paste relevant sections from the manuscript (include quotes in quotation marks "like this" to indicate direct quotes from your manuscript), or elaborate on this item by providing additional information not in the ms, or briefly explain why the item is not applicable/relevant for your study

Your answer

5-vii) Access

Access: Describe how participants accessed the application, in what setting/context, if they had to pay (or were paid) or not, whether they had to be a member of specific group. If known, describe how participants obtained “access to the platform and Internet” [1]. To ensure access for editors/reviewers/readers, consider to provide a “backdoor” login account or demo mode for reviewers/readers to explore the application (also important for archiving purposes, see vi).

	1	2	3	4	5	
subitem not at all important	○	○	○	○	○	essential

Does your paper address subitem 5-vii? *

Copy and paste relevant sections from the manuscript (include quotes in quotation marks "like this" to indicate direct quotes from your manuscript), or elaborate on this item by providing additional information not in the ms, or briefly explain why the item is not applicable/relevant for your study

"Following consent, eligible participants created an account with their email address and a password that they generated to have access to the mobile app and web portal. Participants downloaded the mobile app onto their smartphone. Participants were compensated for completing study questionnaires. "

5-viii) Mode of delivery, features/functionalities/components of the intervention and comparator, and the theoretical framework

Describe mode of delivery, features/functionalities/components of the intervention and comparator, and the theoretical framework [6] used to design them (instructional strategy [1], behaviour change techniques, persuasive features, etc., see e.g., [7, 8] for terminology). This includes an in-depth description of the content (including where it is coming from and who developed it) [1],” whether [and how] it is tailored to individual circumstances and allows users to track their progress and receive feedback” [6]. This also includes a description of communication delivery channels and – if computer-mediated communication is a component – whether communication was synchronous or asynchronous [6]. It also includes information on presentation strategies [1], including page design principles, average amount of text on pages, presence of hyperlinks to other resources, etc. [1].

	1	2	3	4	5	
subitem not at all important	○	○	○	○	○	essential

Does your paper address subitem 5-viii? *

Copy and paste relevant sections from the manuscript (include quotes in quotation marks "like this" to indicate direct quotes from your manuscript), or elaborate on this item by providing additional information not in the ms, or briefly explain why the item is not applicable/relevant for your study

“Interventions
Both Spark and Control programs were divided into five modules to be completed at a pace of one per week over the 5 week intervention period, but participants were able to progress at their own pace. . Content for a given week was not expected to take more than 60 minutes to complete. All participants were prompted to complete a weekly PHQ-8 and a clinical concerns questionnaire in the mobile app. Automated app notification reminders to complete these questionnaires were sent. If users had not opened the app in 3 days, an automated app notification encouraging participants to use the app was sent. Automated reminder notifications were sent 7 days before the end of the intervention period to remind participants that the intervention period would be ending in 7 days.
Crisis resources were available for participants to access anytime in each app.

Spark (Versions 2.1 and 2.2)
The Spark app is based on CBT, which implements behavioral activation [44,45]. A character called ‘Limbot’ is used as a therapeutic guide to encourage users to complete activities and model examples of activities for users. In the app, participants read text, answered questions, inputted text, and completed interactive activities. Participants were encouraged to schedule activities to be completed outside of the app and reflect on the impact on their mood. Tasks in the mobile app progress in a linear fashion-- i.e., each task must be completed to progress to the next task.

Version 2.1 of Spark included a 5 level program focused on providing psychoeducational content and delivering the behavioral activation model of depression by teaching two core skills (mood-activity logging and activity scheduling). Version 2.2 was also divided into 5 levels, and expanded on the content of Version 2.1, adding problem solving, mindfulness, and relapse prevention content. Version 2.2 also included a reward system and animations for completing certain activities, some design changes to the user interface, and an increased number of in-app notifications. The 5 levels of Spark included: Level 1: Onboarding and Introduction to Behavioral Activation; Level 2: Mood tracking; Level 3: Mindfulness and Activity Scheduling; Level 4: Problem Solving and Activity Scheduling; Level 5: Relapse Prevention and Activity Scheduling.
Psychoeducational control
The control app contained 5 modules of age-appropriate psychoeducational content related to the neurobiology of depression and did not contain any active CBT or BA components. In this app, participants read text on screen related to the brain and behavior, the adolescent brain, depression in the brain, neurobiological factors influencing depression, and personality. The same version of the control app was used for the entire study.The 5 levels of the control app included: Lesson 1: Understanding Behavior; Lesson 2: Exploring the Brain; Lesson 3: Mastering Messengers; Lesson 4: Riding the Wave; Lesson 5: People and Personality. “

5-ix) Describe use parameters

Describe use parameters (e.g., intended “doses” and optimal timing for use). Clarify what instructions or recommendations were given to the user, e.g., regarding timing, frequency, heaviness of use, if any, or was the intervention used ad libitum.

	1	2	3	4	5	
subitem not at all important	○	○	○	○	○	essential

Does your paper address subitem 5-ix?

Copy and paste relevant sections from the manuscript (include quotes in quotation marks "like this" to indicate direct quotes from your manuscript), or elaborate on this item by providing additional information not in the ms, or briefly explain why the item is not applicable/relevant for your study

Your answer

5-x) Clarify the level of human involvement

Clarify the level of human involvement (care providers or health professionals, also technical assistance) in the e-intervention or as co-intervention (detail number and expertise of professionals involved, if any, as well as “type of assistance offered, the timing and frequency of the support, how it is initiated, and the medium by which the assistance is delivered”. It may be necessary to distinguish between the level of human involvement required for the trial, and the level of human involvement required for a routine application outside of a RCT setting (discuss under item 21 – generalizability).

	1	2	3	4	5	
subitem not at all important	○	○	○	○	○	essential

Does your paper address subitem 5-x?

Copy and paste relevant sections from the manuscript (include quotes in quotation marks "like this" to indicate direct quotes from your manuscript), or elaborate on this item by providing additional information not in the ms, or briefly explain why the item is not applicable/relevant for your study

Your answer

5-xi) Report any prompts/reminders used

Report any prompts/reminders used: Clarify if there were prompts (letters, emails, phone calls, SMS) to use the application, what triggered them, frequency etc. It may be necessary to distinguish between the level of prompts/reminders required for the trial, and the level of prompts/reminders for a routine application outside of a RCT setting (discuss under item 21 – generalizability).

	1	2	3	4	5	
subitem not at all important	○	○	○	○	○	essential

Does your paper address subitem 5-xi? *

Copy and paste relevant sections from the manuscript (include quotes in quotation marks "like this" to indicate direct quotes from your manuscript), or elaborate on this item by providing additional information not in the ms, or briefly explain why the item is not applicable/relevant for your study

"All participants will be prompted to complete a weekly PHQ-8 and clinical concerns questionnaire in the mobile app. Automated app notification reminders to complete these questionnaires will be sent. If users have not opened the app in 3 days, an automated app notification encouraging participants to use the app will be sent. An automated reminder notification will be sent 7 days before the end of the intervention period to remind participants that the intervention period will be ending in 7 days.

The legal guardian of participants under 18 will be emailed links to complete a parent-reported participant symptom check weekly. If they have not completed the questionnaire, they will be sent a reminder email 6 days later to remind them that the questionnaire is due the next day.

After the 5 week intervention period, participants and their legal guardian (if required) will be emailed links to complete post-intervention self-report assessments via secure web portal. Participants and their legal guardian (if required) will receive automated email reminders to complete the post-intervention assessments after 6 days if they have not completed the assessments. If participants or their legal guardian do not complete the post-intervention assessments within a week the research coordinator may reach out to participants or legal guardians via phone or email to encourage them to complete these assessments. "

5-xii) Describe any co-interventions (incl. training/support)

Describe any co-interventions (incl. training/support): Clearly state any interventions that are provided in addition to the targeted eHealth intervention, as ehealth intervention may not be designed as stand-alone intervention. This includes training sessions and support [1]. It may be necessary to distinguish between the level of training required for the trial, and the level of training for a routine application outside of a RCT setting (discuss under item 21 – generalizability.

	1	2	3	4	5	
subitem not at all important	○	○	○	○	○	essential

Does your paper address subitem 5-xii? *

Copy and paste relevant sections from the manuscript (include quotes in quotation marks "like this" to indicate direct quotes from your manuscript), or elaborate on this item by providing additional information not in the ms, or briefly explain why the item is not applicable/relevant for your study

No co-interventions in this study

6a) Completely defined pre-specified primary and secondary outcome measures, including how and when they were assessed

Does your paper address CONSORT subitem 6a? *

Copy and paste relevant sections from the manuscript (include quotes in quotation marks "like this" to indicate direct quotes from your manuscript), or elaborate on this item by providing additional information not in the ms, or briefly explain why the item is not applicable/relevant for your study

Primary outcome measure: “The primary outcome was a group difference in the change in depression symptoms from baseline to 5-weeks measured by the PHQ-8.”
Secondary Clinical outcomes: “Secondary outcomes included anxiety symptoms (Generalized Anxiety Disorder Scale; GAD-7 [59]), legal guardian-rated depression symptoms of their child (Mood and Feelings Questionnaire; MFQ [61]), participant and legal-guardian (of their child) rated global functioning (Patient-Reported Outcomes Measurement Information System; PROMIS [60]) assessed at baseline and post-intervention. Means, standard deviations, and 95% confidence intervals for the average difference between baseline and post-intervention were computed for these outcomes. A questionnaire was developed internally to assess positive and negative impacts of COVID-19 and the proportion of participants in each group that endorsed each item was computed. “
Safety
Total numbers of AE, ADE, SAE, and UADE were computed per group for all randomized participants.

Other outcomes
Other outcomes measured included program adherence, engagement and acceptability. Program adherence was assessed as the proportion of participants completing all sessions by post-intervention and percent completion per module. Engagement for both groups were assessed by User Engagement Scale (UES-SF) [63], minutes spent in app per week, and total app sessions. Usability was assessed using the System Usability Scale (SUS) [62]. App acceptability was assessed with internally developed self- and legal guardian- reported ratings of the app with a 10-point Likert scale, asking how much the app improved mood or symptoms of depression and how enjoyable it was. Means, standard deviations, and mean differences between groups with 95% confidence intervals were calculated for these measures.

6a-i) Online questionnaires: describe if they were validated for online use and apply CHERRIES items to describe how the questionnaires were designed/deployed

If outcomes were obtained through online questionnaires, describe if they were validated for online use and apply CHERRIES items to describe how the questionnaires were designed/deployed [9].

	1	2	3	4	5	
subitem not at all important	○	○	○	○	○	essential

Does your paper address subitem 6a-i?

Copy and paste relevant sections from manuscript text

Your answer

6a-ii) Describe whether and how “use” (including intensity of use/dosage) was defined/measured/monitored

Describe whether and how “use” (including intensity of use/dosage) was defined/measured/monitored (logins, logfile analysis, etc.). Use/adoption metrics are important process outcomes that should be reported in any ehealth trial.

	1	2	3	4	5	
subitem not at all important	○	○	○	○	○	essential

Does your paper address subitem 6a-ii?

Copy and paste relevant sections from manuscript text

Your answer

6a-iii) Describe whether, how, and when qualitative feedback from participants was obtained

Describe whether, how, and when qualitative feedback from participants was obtained (e.g., through emails, feedback forms, interviews, focus groups).

	1	2	3	4	5	
subitem not at all important	○	○	○	○	○	essential

Does your paper address subitem 6a-iii?

Copy and paste relevant sections from manuscript text

Your answer

6b) Any changes to trial outcomes after the trial commenced, with reasons

Does your paper address CONSORT subitem 6b? *

Copy and paste relevant sections from the manuscript (include quotes in quotation marks "like this" to indicate direct quotes from your manuscript), or elaborate on this item by providing additional information not in the ms, or briefly explain why the item is not applicable/relevant for your study

No changes to trial outcomes after trial commenced

7a) How sample size was determined

NPT: When applicable, details of whether and how the clustering by care provides or centers was addressed

7a-i) Describe whether and how expected attrition was taken into account when calculating the sample size

Describe whether and how expected attrition was taken into account when calculating the sample size.

	1	2	3	4	5	
subitem not at all important	○	○	○	○	○	essential

Does your paper address subitem 7a-i?

Copy and paste relevant sections from manuscript title (include quotes in quotation marks "like this" to indicate direct quotes from your manuscript), or elaborate on this item by providing additional information not in the ms, or briefly explain why the item is not applicable/relevant for your study

Your answer

7b) When applicable, explanation of any interim analyses and stopping guidelines

Does your paper address CONSORT subitem 7b? *

Copy and paste relevant sections from the manuscript (include quotes in quotation marks "like this" to indicate direct quotes from your manuscript), or elaborate on this item by providing additional information not in the ms, or briefly explain why the item is not applicable/relevant for your study

There were no pre-planned interim analyses. Prespecified stopping guidelines included: 1) study recruitment or retention is too low for the study to provide meaningful results; 2) new information becomes available during the trial that necessitates stopping the trial; 3) other situations occur that might warrant stopping the trial. Given that the trial intervention is relatively safe and updates to the mobile app are planned a priori, the trial will not be stopped prior to implementation of Spark version 3 for lack of demonstrated efficacy. The trial may be stopped early if PIs, in collaboration with study clinician and DSMB, determine from interim analyses during implementation of version 3 that the Spark app is not efficacious or if there are safety concerns. The trial will be stopped early if PIs, in collaboration with the study clinician, find that the harm to study participants outweighs the benefit of the scientific evidence to be accrued by continuing the trial at any time.

8a) Method used to generate the random allocation sequence

NPT: When applicable, how care providers were allocated to each trial group

Does your paper address CONSORT subitem 8a? *

Copy and paste relevant sections from the manuscript (include quotes in quotation marks "like this" to indicate direct quotes from your manuscript), or elaborate on this item by providing additional information not in the ms, or briefly explain why the item is not applicable/relevant for your study

Block randomization was implemented ranging in multiples of two from 6-12.

8b) Type of randomisation; details of any restriction (such as blocking and block size)

Does your paper address CONSORT subitem 8b? *

Copy and paste relevant sections from the manuscript (include quotes in quotation marks "like this" to indicate direct quotes from your manuscript), or elaborate on this item by providing additional information not in the ms, or briefly explain why the item is not applicable/relevant for your study

“Participants were randomized 1:1 to the Spark or Control arm using a block randomization approach ranging in multiples of two from 6-12”

9) Mechanism used to implement the random allocation sequence (such as sequentially numbered containers), describing any steps taken to conceal the sequence until interventions were assigned

Does your paper address CONSORT subitem 9? *

Copy and paste relevant sections from the manuscript (include quotes in quotation marks "like this" to indicate direct quotes from your manuscript), or elaborate on this item by providing additional information not in the ms, or briefly explain why the item is not applicable/relevant for your study

Randomization allocation was implemented in the system backend, password protected, and not accessible to study staff. Assignment to a treatment condition happened automatically.

10) Who generated the random allocation sequence, who enrolled participants, and who assigned participants to interventions

Does your paper address CONSORT subitem 10? *

Copy and paste relevant sections from the manuscript (include quotes in quotation marks "like this" to indicate direct quotes from your manuscript), or elaborate on this item by providing additional information not in the ms, or briefly explain why the item is not applicable/relevant for your study

Engineering staff programmed the block randomization procedure into the system backend. Study staff, who did not have access to the randomization procedure, enrolled participants, who were randomly assigned to a treatment condition in the system.

11a) If done, who was blinded after assignment to interventions (for example, participants, care providers, those assessing outcomes) and how

NPT: Whether or not administering co-interventions were blinded to group assignment

11a-i) Specify who was blinded, and who wasn't

Specify who was blinded, and who wasn't. Usually, in web-based trials it is not possible to blind the participants [1, 3] (this should be clearly acknowledged), but it may be possible to blind outcome assessors, those doing data analysis or those administering co-interventions (if any).

	1	2	3	4	5	
subitem not at all important	○	○	○	○	○	essential

Does your paper address subitem 11a-i? *

Copy and paste relevant sections from the manuscript (include quotes in quotation marks "like this" to indicate direct quotes from your manuscript), or elaborate on this item by providing additional information not in the ms, or briefly explain why the item is not applicable/relevant for your study

This was an open-label trial.

11a-ii) Discuss e.g., whether participants knew which intervention was the “intervention of interest” and which one was the “comparator”

Informed consent procedures (4a-ii) can create biases and certain expectations - discuss e.g., whether participants knew which intervention was the “intervention of interest” and which one was the “comparator”.

	1	2	3	4	5	
subitem not at all important	○	○	○	○	○	essential

Does your paper address subitem 11a-ii?

Copy and paste relevant sections from the manuscript (include quotes in quotation marks "like this" to indicate direct quotes from your manuscript), or elaborate on this item by providing additional information not in the ms, or briefly explain why the item is not applicable/relevant for your study

Your answer

11b) If relevant, description of the similarity of interventions

(this item is usually not relevant for ehealth trials as it refers to similarity of a placebo or sham intervention to a active medication/intervention)

Does your paper address CONSORT subitem 11b? *

Copy and paste relevant sections from the manuscript (include quotes in quotation marks "like this" to indicate direct quotes from your manuscript), or elaborate on this item by providing additional information not in the ms, or briefly explain why the item is not applicable/relevant for your study

Not applicable because this was an open label trial with a psychoeducation control

12a) Statistical methods used to compare groups for primary and secondary outcomes

NPT: When applicable, details of whether and how the clustering by care providers or centers was addressed

Does your paper address CONSORT subitem 12a? *

Copy and paste relevant sections from the manuscript (include quotes in quotation marks "like this" to indicate direct quotes from your manuscript), or elaborate on this item by providing additional information not in the ms, or briefly explain why the item is not applicable/relevant for your study

For the primary outcome:

“A linear mixed-effects model (LMM) analysis was implemented on an averaged imputed dataset to evaluate main effects of Group (Spark, Control) and Week (0-5), and the Group x Week interaction using the R lme4 package (v. 1.1.28). Four models were implemented in total including mITT and PP analyses for both Moderate-Severe and Mild-Severe cohorts. In each model, Group and Week were entered as fixed factors. Spark version, and assessment completion days since baseline were included as fixed factors to control for effects of app version and differences in time between completion of successive weekly assessments. PHQ-8 item-level scores and participant were included as random factors for the intercept, and time since baseline assessment was included as a random factor for the slope of participant. FDR-correction was applied to the four p-values for the Group x Week interaction effects. Effect size of the interaction (Cohen’s f2) was computed using Pseudo R2 (f2 =R2/(1- R2))[74,75] with the R MuMIn package (v.1.46.0). Degrees of freedom were estimated using the Satterthwaite method provided in R lmerTest package (v. 3.1-3) [76,77]. Follow-up analyses evaluated the effect of Week within each Group.

We also evaluated whether there was an average minimal clinically important difference (MCID) in symptom severity within each Group, defined as a five point or greater average decrease in PHQ-8 score between baseline and post-intervention [78].

To assess the robustness of the Group x Week interaction, a generalized linear mixed-effect model (GLMM) with MI with the same model specification as the LMM analysis was implemented on item level PHQ-8 data across the 100 imputed datasets using a 2-level PAN method [71] and resulting four p-values were FDR-adjusted.

Group differences in remission rates, defined as PHQ-8<5 at post-intervention [79], and treatment response rates, defined as a 50% reduction in PHQ-8 score between baseline and post-intervention [50] were tested using X2 tests and the resulting four p-values were FDR adjusted. “

For secondary outcomes:
“Means, standard deviations, and 95% confidence intervals for the average difference between baseline and post-intervention were computed for these outcomes. “

12a-i) Imputation techniques to deal with attrition / missing values

Imputation techniques to deal with attrition / missing values: Not all participants will use the intervention/comparator as intended and attrition is typically high in ehealth trials. Specify how participants who did not use the application or dropped out from the trial were treated in the statistical analysis (a complete case analysis is strongly discouraged, and simple imputation techniques such as LOCF may also be problematic [4]).

	1	2	3	4	5	
subitem not at all important	○	○	○	○	○	essential

Does your paper address subitem 12a-i? *

Copy and paste relevant sections from the manuscript (include quotes in quotation marks "like this" to indicate direct quotes from your manuscript), or elaborate on this item by providing additional information not in the ms, or briefly explain why the item is not applicable/relevant for your study

“Missing data analysis
Little’s test [69] was used to determine whether group differences existed in the proportion of missing PHQ-8 data across weeks for both Moderate-Severe and Mild-Severe cohorts. For any resulting significant results, the effects of known factors on missing data including Spark version, treatment group, week, baseline PHQ-8 severity, and age group were evaluated with follow-up X2 tests to determine whether data could be missing at random [70].”

12b) Methods for additional analyses, such as subgroup analyses and adjusted analyses

Does your paper address CONSORT subitem 12b? *

Copy and paste relevant sections from the manuscript (include quotes in quotation marks "like this" to indicate direct quotes from your manuscript), or elaborate on this item by providing additional information not in the ms, or briefly explain why the item is not applicable/relevant for your study

“Post-hoc analyses were also conducted for participants with a baseline PHQ-8≥5 (Mild-Severe Cohort) to evaluate the efficacy of Spark in participants with mild to severe symptoms of depression.”

X26) REB/IRB Approval and Ethical Considerations [recommended as subheading under "Methods"] (not a CONSORT item)

X26-i) Comment on ethics committee approval

	1	2	3	4	5	
subitem not at all important	○	○	○	○	○	essential

Does your paper address subitem X26-i?

Copy and paste relevant sections from the manuscript (include quotes in quotation marks "like this" to indicate direct quotes from your manuscript), or elaborate on this item by providing additional information not in the ms, or briefly explain why the item is not applicable/relevant for your study

Your answer

x26-ii) Outline informed consent procedures

Outline informed consent procedures e.g., if consent was obtained offline or online (how? Checkbox, etc.?), and what information was provided (see 4a-ii). See [6] for some items to be included in informed consent documents.

	1	2	3	4	5	
subitem not at all important	○	○	○	○	○	essential

Does your paper address subitem X26-ii?

Copy and paste relevant sections from the manuscript (include quotes in quotation marks "like this" to indicate direct quotes from your manuscript), or elaborate on this item by providing additional information not in the ms, or briefly explain why the item is not applicable/relevant for your study

Your answer

X26-iii) Safety and security procedures

Safety and security procedures, incl. privacy considerations, and any steps taken to reduce the likelihood or detection of harm (e.g., education and training, availability of a hotline)

	1	2	3	4	5	
subitem not at all important	○	○	○	○	○	essential

Does your paper address subitem X26-iii?

Copy and paste relevant sections from the manuscript (include quotes in quotation marks "like this" to indicate direct quotes from your manuscript), or elaborate on this item by providing additional information not in the ms, or briefly explain why the item is not applicable/relevant for your study

Your answer

RESULTS

13a) For each group, the numbers of participants who were randomly assigned, received intended treatment, and were analysed for the primary outcome
NPT: The number of care providers or centers performing the intervention in each group and the number of patients treated by each care provider in each center

Does your paper address CONSORT subitem 13a? *

Copy and paste relevant sections from the manuscript (include quotes in quotation marks "like this" to indicate direct quotes from your manuscript), or elaborate on this item by providing additional information not in the ms, or briefly explain why the item is not applicable/relevant for your study

“168 adolescents consented to participate in the study. Eight participants did not meet eligibility criteria. 160 were randomized (80/treatment group; Figure 1 for CONSORT diagram; Table 2 for characteristics). 41 participants received Spark Version 2.1 and 39 participants received Spark Version 2.2. Data from participants with a baseline PHQ-8≥10 (Moderate-Severe cohort; Table 2 for characteristics) were included in all planned analyses including mITT (N=121) and PP (N=86). Post-hoc analyses included data from participants with a baseline PHQ≥5 (Mild-Severe cohort; Table 2 for characteristics) for mITT (N=153) and PP (N=109). Three participants skipped a question on a PHQ-8 assessment and were included in PP analyses. The Spark and Control groups did not significantly differ on any of the baseline demographic characteristics (all p’s > 0.05).”

13b) For each group, losses and exclusions after randomisation, together with reasons

Does your paper address CONSORT subitem 13b? (NOTE: Preferably, this is shown in a CONSORT flow diagram) *

Copy and paste relevant sections from the manuscript (include quotes in quotation marks "like this" to indicate direct quotes from your manuscript), or elaborate on this item by providing additional information not in the ms, or briefly explain why the item is not applicable/relevant for your study

“168 adolescents consented to participate in the study. Eight participants did not meet eligibility criteria. 160 were randomized (80/treatment group; Figure 1 for CONSORT diagram; Table 2 for characteristics). 41 participants received Spark Version 2.1 and 39 participants received Spark Version 2.2. Data from participants with a baseline PHQ-8≥10 (Moderate-Severe cohort; Table 2 for characteristics) were included in all planned analyses including mITT (N=121) and PP (N=86). Post-hoc analyses included data from participants with a baseline PHQ≥5 (Mild-Severe cohort; Table 2 for characteristics) for mITT (N=153) and PP (N=109). Three participants skipped a question on a PHQ-8 assessment and were included in PP analyses. The Spark and Control groups did not significantly differ on any of the baseline demographic characteristics (all p’s > 0.05).”

13b-i) Attrition diagram

Strongly recommended: An attrition diagram (e.g., proportion of participants still logging in or using the intervention/comparator in each group plotted over time, similar to a survival curve) or other figures or tables demonstrating usage/dose/engagement.

	1	2	3	4	5	
subitem not at all important	○	○	○	○	○	essential

Does your paper address subitem 13b-i?

Copy and paste relevant sections from the manuscript or cite the figure number if applicable (include quotes in quotation marks "like this" to indicate direct quotes from your manuscript), or elaborate on this item by providing additional information not in the ms, or briefly explain why the item is not applicable/relevant for your study

Your answer

14a) Dates defining the periods of recruitment and follow-up

Does your paper address CONSORT subitem 14a? *

Copy and paste relevant sections from the manuscript (include quotes in quotation marks "like this" to indicate direct quotes from your manuscript), or elaborate on this item by providing additional information not in the ms, or briefly explain why the item is not applicable/relevant for your study

"Recruitment and all data collection were conducted between November 2020 and September 2021"

14a-i) Indicate if critical “secular events” fell into the study period

Indicate if critical “secular events” fell into the study period, e.g., significant changes in Internet resources available or “changes in computer hardware or Internet delivery resources”

	1	2	3	4	5	
subitem not at all important	○	○	○	○	○	essential

Does your paper address subitem 14a-i?

Copy and paste relevant sections from the manuscript (include quotes in quotation marks "like this" to indicate direct quotes from your manuscript), or elaborate on this item by providing additional information not in the ms, or briefly explain why the item is not applicable/relevant for your study

Your answer

14b) Why the trial ended or was stopped (early)

Does your paper address CONSORT subitem 14b? *

Copy and paste relevant sections from the manuscript (include quotes in quotation marks "like this" to indicate direct quotes from your manuscript), or elaborate on this item by providing additional information not in the ms, or briefly explain why the item is not applicable/relevant for your study

Trial ended after recruitment targets were met and all participants had completed data collection (or were lost to follow up).

15) A table showing baseline demographic and clinical characteristics for each group

NPT: When applicable, a description of care providers (case volume, qualification, expertise, etc.) and centers (volume) in each group

Does your paper address CONSORT subitem 15? *

Copy and paste relevant sections from the manuscript (include quotes in quotation marks "like this" to indicate direct quotes from your manuscript), or elaborate on this item by providing additional information not in the ms, or briefly explain why the item is not applicable/relevant for your study

See Table 2 for characteristics

15-i) Report demographics associated with digital divide issues

In ehealth trials it is particularly important to report demographics associated with digital divide issues, such as age, education, gender, social-economic status, computer/Internet/ehealth literacy of the participants, if known.

	1	2	3	4	5	
subitem not at all important	○	○	○	○	○	essential

Does your paper address subitem 15-i? *

Copy and paste relevant sections from the manuscript (include quotes in quotation marks "like this" to indicate direct quotes from your manuscript), or elaborate on this item by providing additional information not in the ms, or briefly explain why the item is not applicable/relevant for your study

See Table 2 for characteristics

16) For each group, number of participants (denominator) included in each analysis and whether the analysis was by original assigned groups

16-i) Report multiple “denominators” and provide definitions

Report multiple “denominators” and provide definitions: Report N’s (and effect sizes) “across a range of study participation [and use] thresholds” [1], e.g., N exposed, N consented, N used more than x times, N used more than y weeks, N participants “used” the intervention/comparator at specific pre-defined time points of interest (in absolute and relative numbers per group). Always clearly define “use” of the intervention.

	1	2	3	4	5	
subitem not at all important	○	○	○	○	○	essential

Does your paper address subitem 16-i? *

Copy and paste relevant sections from the manuscript (include quotes in quotation marks "like this" to indicate direct quotes from your manuscript), or elaborate on this item by providing additional information not in the ms, or briefly explain why the item is not applicable/relevant for your study

168 adolescents consented to participate in the study. Eight participants did not meet eligibility criteria. 160 were randomized (80/treatment group; Figure 1 for CONSORT diagram; Table 2 for characteristics). 41 participants received Spark Version 2.1 and 39 participants received Spark Version 2.2. Data from participants with a baseline PHQ-8≥10 (Moderate-Severe cohort; Table 2 for characteristics) were included in all planned analyses including mITT (N=121) and PP (N=86). Post-hoc analyses included data from participants with a baseline PHQ≥5 (Mild-Severe cohort; Table 2 for characteristics) for mITT (N=153) and PP (N=109). Three participants skipped a question on a PHQ-8 assessment and were included in PP analyses.

16-ii) Primary analysis should be intent-to-treat

Primary analysis should be intent-to-treat, secondary analyses could include comparing only “users”, with the appropriate caveats that this is no longer a randomized sample (see 18-i).

	1	2	3	4	5	
subitem not at all important	○	○	○	○	○	essential

Does your paper address subitem 16-ii?

Copy and paste relevant sections from the manuscript (include quotes in quotation marks "like this" to indicate direct quotes from your manuscript), or elaborate on this item by providing additional information not in the ms, or briefly explain why the item is not applicable/relevant for your study

“An a priori statistical analysis plan was restricted to participants with moderate to severe symptoms at baseline (PHQ-8≥10; Moderate-Severe cohort), unless otherwise specified.” “A modified intention-to-treat (mITT) approach was used for primary analyses by including all participants randomized within the Moderate-Severe cohort. A per protocol (PP) analysis included data from participants in this cohort who had completed all weekly PHQ-8 questionnaires. Post-hoc mITT and PP analyses were also conducted for participants with a baseline PHQ-8≥5 (Mild-Severe Cohort) to evaluate the efficacy of Spark in participants with mild to severe symptoms of depression.”

17a) For each primary and secondary outcome, results for each group, and the estimated effect size and its precision (such as 95% confidence interval)

Does your paper address CONSORT subitem 17a? *

Copy and paste relevant sections from the manuscript (include quotes in quotation marks "like this" to indicate direct quotes from your manuscript), or elaborate on this item by providing additional information not in the ms, or briefly explain why the item is not applicable/relevant for your study

Primary outcome: Moderate-Severe cohort. Also presented in Table 3
Modified Intention To Treat. There was a non-significant GroupxWeek interaction, t127.66=-1.911, P=.06 (FDR adj. P=.06) (Figure 2a). There was a significant effect of Week, t1759.08=-3.508, P<.001 in the Spark group (change score=-5.08 [95%CI: -6.72; -3.42]), and in the Control group, t561.98=-3.244, P=.001 (change score=-3.51 [95%CI: -5.09; -1.93]).

At the end of the intervention period, the Spark group showed significantly higher remission rates compared to Control (Spark=17%;Control=3%;X21=6.183, P=.01; FDR adj P=.03). Treatment response rates between groups (Spark =24%; Control=14%) were not statistically significant (X21=1.968, P=.07; FDR adj. P=.16).

Secondary Clinical outcomes: “Means, standard deviations, and 95% confidence intervals of the difference between baseline and post-intervention scores for all secondary participant- and legal-guardian-reported measures are reported per group in Table 5. “

17a-i) Presentation of process outcomes such as metrics of use and intensity of use

In addition to primary/secondary (clinical) outcomes, the presentation of process outcomes such as metrics of use and intensity of use (dose, exposure) and their operational definitions is critical. This does not only refer to metrics of attrition (13-b) (often a binary variable), but also to more continuous exposure metrics such as “average session length”. These must be accompanied by a technical description how a metric like a “session” is defined (e.g., timeout after idle time) [1] (report under item 6a).

	1	2	3	4	5	
subitem not at all important	○	○	○	○	○	essential

Does your paper address subitem 17a-i?

Copy and paste relevant sections from the manuscript (include quotes in quotation marks "like this" to indicate direct quotes from your manuscript), or elaborate on this item by providing additional information not in the ms, or briefly explain why the item is not applicable/relevant for your study

Your answer

17b) For binary outcomes, presentation of both absolute and relative effect sizes is recommended

Does your paper address CONSORT subitem 17b? *

Copy and paste relevant sections from the manuscript (include quotes in quotation marks "like this" to indicate direct quotes from your manuscript), or elaborate on this item by providing additional information not in the ms, or briefly explain why the item is not applicable/relevant for your study

Not applicable because we did not have binary outcomes

18) Results of any other analyses performed, including subgroup analyses and adjusted analyses, distinguishing pre-specified from exploratory

Does your paper address CONSORT subitem 18? *

Copy and paste relevant sections from the manuscript (include quotes in quotation marks "like this" to indicate direct quotes from your manuscript), or elaborate on this item by providing additional information not in the ms, or briefly explain why the item is not applicable/relevant for your study

“Mild-Severe cohort
Modified Intention To Treat. There was a significant GroupxWeek interaction, t160.20=-3.054, P=.003 (FDR adj. P=.005) (Figure 2c). There was a significant effect of Week in the Spark group, t1697.40=-3.32, p<.001 (change score=-4.85 [95%CI: -6.59; -3.09), but not in the Control group, t1177.40=-1.838, P=.07, (change score=-2.86 [95%CI: -4.43; -1.30]).

At the end of the intervention period, the Spark group showed significantly higher remission (Spark=27%; Control=11%; X21=6.08, P=.01; FDR adj. P=.03) and treatment response (Spark=30%; Control=15%; X21=4.674, P=.03; FDR adj. P=.04) rates compared to Control. "

18-i) Subgroup analysis of comparing only users

A subgroup analysis of comparing only users is not uncommon in ehealth trials, but if done, it must be stressed that this is a self-selected sample and no longer an unbiased sample from a randomized trial (see 16-iii).

	1	2	3	4	5	
subitem not at all important	○	○	○	○	○	essential

Does your paper address subitem 18-i?

Copy and paste relevant sections from the manuscript (include quotes in quotation marks "like this" to indicate direct quotes from your manuscript), or elaborate on this item by providing additional information not in the ms, or briefly explain why the item is not applicable/relevant for your study

Your answer

19) All important harms or unintended effects in each group

(for specific guidance see CONSORT for harms)

Does your paper address CONSORT subitem 19? *

Copy and paste relevant sections from the manuscript (include quotes in quotation marks "like this" to indicate direct quotes from your manuscript), or elaborate on this item by providing additional information not in the ms, or briefly explain why the item is not applicable/relevant for your study

“There were no serious adverse events or adverse device effects in either group. There were a total of two adverse events in the Control group and four in the Spark group. Both adverse events in the Control group were clinical deteriorations in PHQ-8 scores for two consecutive weeks. In the Spark group, there was one clinical deterioration in PHQ-8 score for two consecutive weeks, two instances of participant-reported suicidal ideation, and one instance of a legal guardian reporting that the participant was experiencing non-suicidal self-injury ideation.”

19-i) Include privacy breaches, technical problems

Include privacy breaches, technical problems. This does not only include physical “harm” to participants, but also incidents such as perceived or real privacy breaches [1], technical problems, and other unexpected/unintended incidents. “Unintended effects” also includes unintended positive effects [2].

	1	2	3	4	5	
subitem not at all important	○	○	○	○	○	essential

Does your paper address subitem 19-i?

Copy and paste relevant sections from the manuscript (include quotes in quotation marks "like this" to indicate direct quotes from your manuscript), or elaborate on this item by providing additional information not in the ms, or briefly explain why the item is not applicable/relevant for your study

Your answer

19-ii) Include qualitative feedback from participants or observations from staff/researchers

Include qualitative feedback from participants or observations from staff/researchers, if available, on strengths and shortcomings of the application, especially if they point to unintended/unexpected effects or uses. This includes (if available) reasons for why people did or did not use the application as intended by the developers.

	1	2	3	4	5	
subitem not at all important	○	○	○	○	○	essential

Does your paper address subitem 19-ii?

Copy and paste relevant sections from the manuscript (include quotes in quotation marks "like this" to indicate direct quotes from your manuscript), or elaborate on this item by providing additional information not in the ms, or briefly explain why the item is not applicable/relevant for your study

Your answer

DISCUSSION

22) Interpretation consistent with results, balancing benefits and harms, and considering other relevant evidence

NPT: In addition, take into account the choice of the comparator, lack of or partial blinding, and unequal expertise of care providers or centers in each group

22-i) Restate study questions and summarize the answers suggested by the data, starting with primary outcomes and process outcomes (use)

Restate study questions and summarize the answers suggested by the data, starting with primary outcomes and process outcomes (use).

	1	2	3	4	5	
subitem not at all important	○	○	○	○	○	essential

Does your paper address subitem 22-i? *

Copy and paste relevant sections from the manuscript (include quotes in quotation marks "like this" to indicate direct quotes from your manuscript), or elaborate on this item by providing additional information not in the ms, or briefly explain why the item is not applicable/relevant for your study

“This RCT showed that, in planned and exploratory analyses, Spark was efficacious in treating mild-severe symptoms of depression including a clinically meaningful reduction in depression symptoms, and a significantly larger reduction in depression symptoms, as well as higher remission and treatment response rates at post intervention compared to an active control. Spark did not negatively impact participant safety, with no adverse device effects reported. “

22-ii) Highlight unanswered new questions, suggest future research

Highlight unanswered new questions, suggest future research.

	1	2	3	4	5	
subitem not at all important	○	○	○	○	○	essential

Does your paper address subitem 22-ii?

Copy and paste relevant sections from the manuscript (include quotes in quotation marks "like this" to indicate direct quotes from your manuscript), or elaborate on this item by providing additional information not in the ms, or briefly explain why the item is not applicable/relevant for your study

Your answer

20) Trial limitations, addressing sources of potential bias, imprecision, and, if relevant, multiplicity of analyses

20-i) Typical limitations in ehealth trials

Typical limitations in ehealth trials: Participants in ehealth trials are rarely blinded. Ehealth trials often look at a multiplicity of outcomes, increasing risk for a Type I error. Discuss biases due to non-use of the intervention/usability issues, biases through informed consent procedures, unexpected events.

	1	2	3	4	5	
subitem not at all important	○	○	○	○	○	essential

Does your paper address subitem 20-i? *

Copy and paste relevant sections from the manuscript (include quotes in quotation marks "like this" to indicate direct quotes from your manuscript), or elaborate on this item by providing additional information not in the ms, or briefly explain why the item is not applicable/relevant for your study

“The study was not statistically powered to detect group differences in PHQ-8 symptom change, therefore, these results should be replicated in a fully powered sample. Powered analyses are also warranted to directly investigate impacts of engagement, baseline symptoms severity, and concurrent treatments on outcomes. Although an active control was used, the open-label design of the study is a limitation as participants were not blinded to treatment assignment and the primary outcome was a self-report PHQ-8; blinded studies with clinician reported outcomes will provide stronger evidence of efficacy. Exploratory results including adolescents with mild to severe symptoms at baseline, while promising, should be interpreted with caution and replicated. Similarly, while our PP analysis indicates that engagement with Spark may drive symptom improvement, these observations may be due to survivorship bias. The eligibility criteria for this study were intentionally inclusive to allow adolescents who were seeking resources during the pandemic to participate and did not require stability on concurrent treatment, an assessment for treatment-resistance, or stratify groups based on concurrent treatment, which may have introduced noise into the data. “

21) Generalisability (external validity, applicability) of the trial findings

NPT: External validity of the trial findings according to the intervention, comparators, patients, and care providers or centers involved in the trial

21-i) Generalizability to other populations

Generalizability to other populations: In particular, discuss generalizability to a general Internet population, outside of a RCT setting, and general patient population, including applicability of the study results for other organizations

	1	2	3	4	5	
subitem not at all important	○	○	○	○	○	essential

Does your paper address subitem 21-i?

Copy and paste relevant sections from the manuscript (include quotes in quotation marks "like this" to indicate direct quotes from your manuscript), or elaborate on this item by providing additional information not in the ms, or briefly explain why the item is not applicable/relevant for your study

Your answer

21-ii) Discuss if there were elements in the RCT that would be different in a routine application setting

Discuss if there were elements in the RCT that would be different in a routine application setting (e.g., prompts/reminders, more human involvement, training sessions or other co-interventions) and what impact the omission of these elements could have on use, adoption, or outcomes if the intervention is applied outside of a RCT setting.

	1	2	3	4	5	
subitem not at all important	○	○	○	○	○	essential

Does your paper address subitem 21-ii?

Copy and paste relevant sections from the manuscript (include quotes in quotation marks "like this" to indicate direct quotes from your manuscript), or elaborate on this item by providing additional information not in the ms, or briefly explain why the item is not applicable/relevant for your study

Your answer

OTHER INFORMATION

23) Registration number and name of trial registry

Does your paper address CONSORT subitem 23? *

Copy and paste relevant sections from the manuscript (include quotes in quotation marks "like this" to indicate direct quotes from your manuscript), or elaborate on this item by providing additional information not in the ms, or briefly explain why the item is not applicable/relevant for your study

ClinicalTrials.gov Identifier: NCT04524598

24) Where the full trial protocol can be accessed, if available

Does your paper address CONSORT subitem 24? *

Cite a Multimedia Appendix, other reference, or copy and paste relevant sections from the manuscript (include quotes in quotation marks "like this" to indicate direct quotes from your manuscript), or elaborate on this item by providing additional information not in the ms, or briefly explain why the item is not applicable/relevant for your study

ClinicalTrials.gov Identifier: NCT04524598

25) Sources of funding and other support (such as supply of drugs), role of funders

Does your paper address CONSORT subitem 25? *

Copy and paste relevant sections from the manuscript (include quotes in quotation marks "like this" to indicate direct quotes from your manuscript), or elaborate on this item by providing additional information not in the ms, or briefly explain why the item is not applicable/relevant for your study

“This study was supported with funding from Limbix Health, Inc. and a Small Business Innovation Research grant (R44MH12563) from the National Institutes of Health.”

X27) Conflicts of Interest (not a CONSORT item)

X27-i) State the relation of the study team towards the system being evaluated

In addition to the usual declaration of interests (financial or otherwise), also state the relation of the study team towards the system being evaluated, i.e., state if the authors/evaluators are distinct from or identical with the developers/sponsors of the intervention.

	1	2	3	4	5	
subitem not at all important	○	○	○	○	○	essential

Does your paper address subitem X27-i?

Copy and paste relevant sections from the manuscript (include quotes in quotation marks "like this" to indicate direct quotes from your manuscript), or elaborate on this item by providing additional information not in the ms, or briefly explain why the item is not applicable/relevant for your study

Your answer

About the CONSORT EHEALTH checklist

As a result of using this checklist, did you make changes in your manuscript? *

- ○ yes, major changes
- ○ yes, minor changes
- ◉ no

What were the most important changes you made as a result of using this checklist?

Your answer

How much time did you spend on going through the checklist INCLUDING making changes in your manuscript *

I spent 4 hours completing this form

As a result of using this checklist, do you think your manuscript has improved? *

- ○ yes
- ○ no
- ◉ Other: We did not make any changes

Would you like to become involved in the CONSORT EHEALTH group?

This would involve for example becoming involved in participating in a workshop and writing an "Explanation and Elaboration" document

- ○ yes
- ◉ no
- ○ Other:

Clear selection

Any other comments or questions on CONSORT EHEALTH

Your answer

STOP - Save this form as PDF before you click submit

To generate a record that you filled in this form, we recommend to generate a PDF of this page (on a Mac, simply select "print" and then select "print as PDF") before you submit it.

When you submit your (revised) paper to JMIR, please upload the PDF as supplementary file.

Don't worry if some text in the textboxes is cut off, as we still have the complete information in our database. Thank you!

Final step: Click submit !

Click submit so we have your answers in our database!

Submit Clear form

Never submit passwords through Google Forms.

This form was created outside of your domain. Report Abuse - Terms of Service - Privacy Policy

Google Forms
